# Synthesis and Antibacterial Activity of Metal(loid) Nanostructures by Environmental Multi-Metal(loid) Resistant Bacteria and Metal(loid)-Reducing Flavoproteins

**DOI:** 10.3389/fmicb.2018.00959

**Published:** 2018-05-15

**Authors:** Maximiliano Figueroa, Valentina Fernandez, Mauricio Arenas-Salinas, Diego Ahumada, Claudia Muñoz-Villagrán, Fabián Cornejo, Esteban Vargas, Mauricio Latorre, Eduardo Morales, Claudio Vásquez, Felipe Arenas

**Affiliations:** ^1^Laboratorio Microbiología Molecular, Departamento de Biología, Facultad de Química y Biología, Universidad de Santiago de Chile, Santiago, Chile; ^2^Centro de Bioinformática y Simulación Molecular, Universidad de Talca, Talca, Chile; ^3^Departamento de Ciencias Básicas, Facultad de Ciencia, Universidad Santo Tomas, Sede Santiago, Chile; ^4^Center for the Development of Nanoscience and Nanotechnology, Santiago, Chile; ^5^Mathomics, Centro de Modelamiento Matemático, Universidad de Chile, Beauchef, Santiago, Chile; ^6^Fondap-Center of Genome Regulation, Facultad de Ciencias, Universidad de Chile, Santiago, Chile; ^7^Laboratorio de Bioinformática y Expresión Génica, INTA, Universidad de Chile, Santiago, Chile; ^8^Instituto de Ciencias de la Ingeniería, Universidad de O'Higgins, Rancagua, Chile; ^9^uBiome, San Francisco, CA, United States

**Keywords:** metal, metalloid, reduction, resistance, environmental bacteria, flavoprotein, nanostructure, bioremediation

## Abstract

Microbes are suitable candidates to recover and decontaminate different environments from soluble metal ions, either via reduction or precipitation to generate insoluble, non-toxic derivatives. In general, microorganisms reduce toxic metal ions generating nanostructures (NS), which display great applicability in biotechnological processes. Since the molecular bases of bacterial reduction are still unknown, the search for new -environmentally safe and less expensive- methods to synthesize NS have made biological systems attractive candidates. Here, 47 microorganisms isolated from a number of environmental samples were analyzed for their tolerance or sensitivity to 19 metal(loid)s. Ten of them were highly tolerant to some of them and were assessed for their ability to reduce these toxicants *in vitro*. All isolates were analyzed by 16S rRNA gene sequencing, fatty acids composition, biochemical tests and electron microscopy. Results showed that they belong to the *Enterobacter, Staphylococcus, Acinetobacter*, and *Exiguobacterium* genera. Most strains displayed metal(loid)-reducing activity using either NADH or NADPH as cofactor. While *Acinetobacter schindleri* showed the highest tellurite (${\text{TeO}}_{3}^{2-}$) and tetrachloro aurate (${\text{AuCl}}_{4}^{-}$) reducing activity, *Staphylococcus sciuri* and *Exiguobacterium acetylicum* exhibited selenite (${\text{SeO}}_{3}^{2-}$) and silver (Ag^+^) reducing activity, respectively. Based on these results, we used these bacteria to synthetize, *in vivo and in vitro* Te, Se, Au, and Ag-containing nanostructures. On the other hand, we also used purified *E. cloacae* glutathione reductase to synthesize *in vitro* Te-, Ag-, and Se-containing NS, whose morphology, size, composition, and chemical composition were evaluated. Finally, we assessed the putative anti-bacterial activity exhibited by the *in vitro* synthesized NS: Te-containing NS were more effective than Au-NS in inhibiting *Escherichia coli* and *Listeria monocytogenes* growth. Aerobically synthesized TeNS using MF09 crude extracts showed MICs of 45- and 66- μg/ml for *E. coli* and *L. monocytogenes*, respectively. Similar MIC values (40 and 82 μg/ml, respectively) were observed for TeNS generated using crude extracts from *gorA*-overexpressing *E. coli*. In turn, AuNS MICs for *E. coli* and *L. monocytogenes* were 64- and 68- μg/ml, respectively.

## Introduction

More than half of the elements in the Periodic Table are represented by metals and metalloids. Whereas some of them are essential for different cellular processes, including electron transfer, enzyme catalysis, and stabilization of protein structure, others such as Ag^+^, Hg^+^, ${\text{SeO}}_{4}^{2-}$, and ${\text{TeO}}_{4}^{2-}$ are highly toxic even at very low concentrations (Pérez et al., 2007; Zannoni et al., 2008; Lemire et al., 2013). Thus, organisms like bacteria must maintain a strict balance of the intracellular concentration of these metal(loid)s. Under aerobic conditions the toxicity of compounds containing these elements is mainly related to the production of reactive oxygen species (ROS), which can attack key cell components including polyunsaturated fatty acids, proteins, and nucleic acids (Borsetti et al., 2005; Tremaroli et al., 2007; Vrionis et al., 2015). Additionally, protein carbonylation (Contreras and Vásquez, 2010), [4Fe-4S] cluster dismantling of iron-sulfur proteins (Calderón et al., 2009), membrane lipid peroxidation (Lemire et al., 2013; Pradenas et al., 2013), blocking of cysteine and methionine residues (Stolz and Oremland, 1999), increased cell permeability, and the interference of metabolic pathways have also been observed (Carotti et al., 2000).

Given their great biotechnological potential, we focused our interest in characterizing NS containing silver, gold or the metalloids tellurium and selenium. Selenium can be found in the environment as a solid [Se (0), Se^0^], forming part of selenium-containing amino acids, and as the selenium oxyanions selenate [Se (VI), ${\text{SeO}}_{4}^{2-}$] and the most toxic, selenite [Se (IV), ${\text{SeO}}_{3}^{2-}$] (Stolz and Oremland, 1999). Conversely to selenium, tellurium (Te) has no known biological role to date (Ba et al., 2010) and given its metallic characteristics, it is found in various redox states, mainly elemental tellurium [Te (0), Te^0^], telluride [Te (II), Te^2−^], tellurite [Te (IV), ${\text{TeO}}_{3}^{2-}$], and tellurate [Te (VI), ${\text{TeO}}_{4}^{2-}$], both oxyanions being highly toxic to most organisms (Nies and Silver, 1995; Taylor, 1999). In turn, silver is mainly found in the 1+ oxidation state [Ag (I)], and less commonly as [Ag (II)], [Ag (III)], or metallic silver (Ag^0^) (Burriel et al., 2006). Finally, gold is found as metallic gold (Au^0^) as well as in six other oxidation states, 1+ [Au (I)] and 3+ [Au (III)] being the most commonly found in nature (Nies and Grass, 2009).

To date, a number of genetic and biochemical mechanisms that microbial systems use to cope with the toxicity of defined metal(loid)s are relatively well known; however, a strategy providing universal resistance to all these toxicants has not been reported so far. In general, one of the most common strategies employed by microbes to tolerate high metal(loid) concentrations, is their chemical modification to less-toxic forms (Silver and Phung, 2005). For instance, this kind of resistance mechanisms have been observed in *Rhodobacter capsulatus* (Turner et al., 2011), *Geobacillus* sp. (Correa-Llantén et al., 2013), and *Shewanella oneidensis* (Klonowska et al., 2005) which reduce Te (IV), Au (III) and Se (IV), respectively.

Metal(loid) reduction by microbial systems could be of great help in decontaminating soluble metal ions from soils, forming insoluble, less-toxic derivatives that often generate metal arrangements at the nanoscale (Narayanan and Sakthivel, 2010; Thakkar et al., 2010), also known as nanostructures (NS) exhibiting one or more dimensions <100 nm. Gold (Correa-Llantén et al., 2013), selenium (Song et al., 2017), silver (Gurunathan et al., 2009), tellurium (Arenas-Salinas et al., 2016), and uranium (Suresh, 2012) NS have been reported, opening a great interest because of their potential application in the field of biotechnology.

A number of flavoproteins able of metal ion reduction to less toxic, insoluble forms have been described, including (i) mercury reductase (MerA) (Silver and Phung, 2005), (ii) glutathione reductase (Au^3+^ and ${\text{TeO}}_{3}^{2-}$ reduction, Pugin et al., 2014), (iii) fumarate reductase (selenite reduction, Song et al., 2017), (iv) thioredoxin reductase and alkyl hydroperoxide reductase that reduce tellurite (Arenas-Salinas et al., 2016) and (v) nitrate reductase, able to reduce tellurite as well as silver (Kumar et al., 2007).

Because the optical, mechanical, and fluorescent properties of metal(loid)-containing NS are different from those exhibited by matter at larger scales (Suresh, 2012), NS display a wide range of applications in physics, chemistry, electronics, optics, materials science, biomedicine, and agriculture (Thakkar et al., 2010). The fast development of nanotechnology has provided several kinds of materials for biomedical applications, including cancer detection therapies (Rao, 2008), and antibacterial treatments (Amstad et al., 2011; Azam et al., 2012). In fact, the use of metallic NS displaying bactericidal activity has been reported (Sharma et al., 2009; Li et al., 2011; Cui et al., 2012; Xiu et al., 2012; Dizaj et al., 2014; Pugin et al., 2014; Oves et al., 2017). In other studies, gold-, tellurium-, copper -, silver-, zinc-, and titanium-containing NS proved to be effective as antibacterials against *Escherichia coli* (Cioffi and Rai, 2012).

Summarizing, since NS production by chemical methods often involve high temperature, anaerobic conditions and toxic reagents that in the end affect the clinical application of nanostructures, the generation and characterization of new metallic NS by biological systems is important either for basic as well as for applied research. In this general context, the search for new environmentally friendly and inexpensive methods for metal(loid)-containing NS generation has made biological systems desirable candidates for both *in vivo* and *in vitro* NS production, either using whole microorganisms or purified enzymes.

## Materials and methods

### Isolation and selection of metal(loid)-resistant bacteria

Twenty-two samples were collected from two different Chilean regions from which bacterial isolates were obtained: (i) north (Blanca, Chaxa and Céjar lagoons, El Tatío geyser, Death Valley) and (ii) mid-south (Maule lagoon, El Teniente copper mine). A sample from Uyuni Salar, Bolivia, was also included in this study. In turn, isolates MF02 and MF05 were kindly provided by Dr. Juan Carlos Tantaléan, Universidad San Luis Gonzaga, Ica, Perú.

Four different culture media were individually inoculated with each sample and incubated at 25 or 37°C for 24 h: LB (Sambrook and Russell, 2001); R2A (0.5 g/L yeast extract, 0.5 g/L peptone, 0.5 g/L casamino acids, 0.5 g/L glucose, 0.5 g/L soluble starch, 0.3 g/L sodium pyruvate, 0.3 g/L K_2_HPO_4_, and 0.5 g/L MgSO_4_ adjusted to pH 7.2); Acidiphilium (2 g/L (NH_4_)_2_SO_4_, 0.1 g/L KCl, 0.5 g/L K_2_HPO_4_, 0.5 g/L MgSO_4_, 0.3 g/L yeast extract and 1 g/L D-glucose adjusted to pH 3.0); and Aciduric thermophilic *Bacillus* strains (ATB) (0.2 g/L (NH_4_)_2_SO_4_, 0.5 g/L MgSO_4_, 0.25 g/L CaCl_2_, 3 g/L KH_2_PO_4_, 1 g/L yeast extract, 1 g/L tryptone, 1 g/L glucose and 1 g/L starch adjusted to pH 4.3). Pure cultures were obtained after sequential dilutions and successive passages using appropriate solid media.

Purified colonies were seeded on LB-agar plates that contained different concentrations of the following compounds: K_2_TeO_3_ (0.04–0.4 mM), K_2_CrO_4_ (1–10 mM), CdCl_2_ (3–5 mM), CoCl_2_ (3–5 mM), CuSO_4_ (3–5 mM), AgNO_3_ (1–5 mM), Al(SO_4_)_3_ (5 mM), FeCl_3_ (5–10 mM), PbCl_2_ (3 mM), UO_2_(CH_3_COO)_2_ (2–10 mM), ZnSO_4_ (2–5 mM), NiSO_4_ (2–5 mM), MnSO_4_ (10–20 mM), HgCl_2_ (0.02–0.1 mM), Na_2_SeO_3_ (10–50 mM), FeCl_2_ (5–10 mM), NaAsO_2_ (5–10 mM), Na_2_HAsO_4_ (10–50 mM), or HAuCl_4_ (0.02–1 mM). Selection was for bacterial growth at the highest concentrations and when applicable, also by observing metal(loid) reduction. Strains MF01, MF03, MF04, and MF08 were from Maule lagoon, MF06 from Blanca lagoon (Atacama, Chile), MF07 from Death Valley and MF09 from El Teniente copper mine. Isolate MF10 was from Uyuni Salar, Bolivia.

### Growth, identification, and characterization of the environmental strains

Cells were grown in LB medium at 37°C; *E. coli* BL21 carrying the pET 101/*gorA* vector was grown in ampicillin (0.1 mg/ml)-supplemented LB medium. This *gorA*-expressing plasmid was constructed as follows: the *gorA* gene was amplified from the environmental strain MF01 using primers CACCATGACTAAGCATTATGACTACATCGCA (Gforward) and ACGCATGGTGACAAATTCTTCG (Greverse). PCR products were inserted into the vector Champion TM pET101 Directional TOPO Expression (Invitrogen®). Correct gene insertion was checked by PCR using primers pETForward (ATGCGTCCGGCGTAGAGG) and pETReverse (GCTAGTTATTGCTCAGCGGTGG). Their identity was confirmed by DNA sequencing. The expression of the cloned gene was induced with 1 mM IPTG for 16 h. GorA was purified by Ni-affinity chromatography (HisTrap HP, GE Healthcare).

Physiological characterization of the isolates was carried out using standard biochemical assays (API ZYM and API 20E; BioMérieux Inc.), which were conducted according to the manufacturer's instructions. Morphological analyses were initially done by Gram staining and in the case of selected strains, also by scanning electron microscopy (SEM) as described by Arenas et al. (2014a). Briefly, samples were visualized and analyzed by low voltage electron microscopy using a LVEM5 Benchtop equipment (Center for Bioinformatics and Molecular Simulation).

Bacterial identification was accomplished by sequencing the 16S rRNA gene using the universal primer 8F (forward) and 1492R (reverse). GeneBank accession numbers for the 16S rRNA nucleotide sequences are: MG461635 (MF01), MG459178 (MF02), MG461634 (MF03), MG461632 (MF04), MG461629 (MF05), MG461631 (MF06), MG461630 (MF07), MG461633 (MF08), MG461636 (MF09), and MG459165 (MF10).

Phylogenetic trees were constructed using the MEGA 6.06 program applying the *neighbor-joining* method (Saitou and Nei, 1987), while distances were calculated with bootstrapping (100) applying the *Tamura-Nei* method (Tamura et al., 2004). Bacterial identity was also assessed by analyzing the fatty acid composition at the DSMZ Institute (Braunschweig, Germany).

Determination of minimal inhibitory concentrations (MIC) was carried out using serial dilutions (1: 2) of sterile solutions of K_2_TeO_3_, AgNO_3_, HAuCl_4_, K_2_CrO_4_, CdCl_2_, NaAsO_2_, CuSO_4_ and Na_2_SeO_3_ in 1 ml of LB medium in 48-well plates. Subsequently, 10 μl of cultures grown in LB medium up to OD_600_ ~0.6 were added to each well and incubation proceeded with constant shaking at the optimal growth temperature of the particular isolate. MICs were determined after 24 h of incubation. For growth curve construction, overnight cultures were diluted 1:100 with fresh LB medium and incubated with shaking up to OD_600_ ~ 0.6. Then, 10 μL were added to 1 mL of fresh LB medium containing 4 μM K_2_TeO_3_, 15 μM AgNO_3_ or HAuCl_4_. Bacterial growth was monitored at 600 nm every 30 min for 14 h in a TECAN Infinite M200 Pro multimode plate reader.

### Determination of enzymatic activity

Metal(loid)-reducing activity was determined in a final volume of 200 μl which contained 50 mM Tris-HCl buffer pH 8.0 (or 9.0), 50 mM potassium phosphate pH 7.0, 1 mM NADH (or NADPH), 1 mM β-mercaptoethanol and the crude extract; metal(loid)s tested for reduction were ${\text{TeO}}_{3}^{2-}$ (1 mM), ${\text{SeO}}_{3}^{2-}$ (1 mM), AgNO_3_ (0.2 mM) and HAuCl_4_ (1 mM). Reactions were monitored in a TECAN Infinite M200 Pro multimode plate reader at the wavelength that the metal(loid) absorbs in its elemental state: 500 nm (Te; Pugin et al., 2014), 400 nm (Se; Song et al., 2017), 424 nm (Ag; Kumar et al., 2007), and 540 nm (Au; Correa-Llantén et al., 2013). The optimal buffer and best nicotinamide cofactor were determined for each crude extract; an enzyme unit (U) was defined as the amount of enzyme required to increase the absorbance by 0.001 units/min at the respective wavelength. Metal(loid)-reducing activity was also assessed by native polyacrylamide gel electrophoresis (native PAGE). Briefly, proteins in crude extracts were fractionated in 10% gels; after the run, activity was revealed by submerging the gel in the appropriate buffer containing the metal(loid) and a mixture of 1 mM NADH/NADPH as electron donor.

### *In vivo* and *in vitro* synthesis of nanostructures

The isolates selected for synthesizing NS *in vivo* were grown to exponential phase (OD_600_ ~ 0.5) and treated with ¼ of the metal(loid) MIC (${\text{TeO}}_{3}^{2-}$, ${\text{SeO}}_{3}^{2-}$, Ag^+^ or ${\text{AuCl}}_{4}^{-}$), incubated for 4 h and then centrifuged for 10 min at 9,000 *g*. The bacterial pellet was sent for thin sectioning and transmission electron microscopy (TEM) analysis (Advanced Microscopy Unit (AMU), at Pontificia Universidad Católica de Chile).

Formation of NS *in vitro* was carried out using 150 μg/ml of purified recombinant glutathione reductase (GorA) from *E. cloacae* (isolate MF01). Additionally, crude extracts that contained GorA overexpressed for 2 h were used with reduction buffer (50 mM potassium phosphate pH 7.0, 1 mM NADPH, 1 mM β-mercaptoethanol and 0.15 mM ${\text{TeO}}_{3}^{2-}$ or 1 mM ${\text{SeO}}_{3}^{2-}$). In addition, crude extracts (200 μg/ml of protein per reaction) from strains of interest were used for the production of NS by incubation with 1 mM Ag^+^, AuCl_4_, ${\text{TeO}}_{3}^{2-}$ or 10 mM ${\text{SeO}}_{3}^{2-}$ for 16 h in reduction buffer under optimal pH conditions and the preferred nicotinamide cofactor.

### NS characterization

A drop containing *in vitro* generated NS was placed on a copper grid and NS morphology was analyzed by TEM using a Hitachi Transmission Electron Microscope HT7700. Their chemical composition was determined through X-ray energy dispersion spectroscopy (EDS) using a SEM Zeiss EVO MA 10, EDS Penta FET Precision X-act Oxford Instruments. Both studies were conducted at the Center for the Development of Nanoscience and Nanotechnology–CEDENNA, USACH. In turn, the hydrodynamic diameter of the metallic nanostructures was assessed by dynamic light scattering (DLS) using a Marlvern Zetasizer Nano ZS equipment at room temperature (25°C) as described by Arenas-Salinas et al. (2016). Final values were calculated based on three independent measurements of twenty repetitions. Furthermore, metal(loid)s were quantified using total reflection X-ray fluorescence spectrophotometry (TXRF). Briefly, *in vitro*-synthesized NS were centrifuged at 13,000 × *g* for 90 min and the sediment was suspended in 200 μl of 65% HNO_3_. Samples were heated at 60°C ~2 h and analyzed in a TXRF Bruker S2 PICOFOX Spectrometer at Bioinformatics and Gene Expression laboratory of the Institute of Nutrition and Food Technology (INTA, University of Chile).

### NS antibacterial activity

A suspension of *in vitro* synthesized NS was used to challenge *Escherichia coli* and *Listeria monocytogenes*. The NS were dialyzed against 50 mM Tris-HCl buffer (pH 8.0 or 9.0) or 50 mM potassium phosphate (pH 7.0), accordingly to the buffer used to generate them. Dialysis was for 4 h at room temperature with constant shaking. The dialyzed material was maintained at 4°C until further analysis. Concentrations of these metallic NS were calculated by determining the dry weight which was expressed in μg/ml. MIC determinations of the dialyzed NS and growth curves of *E. coli* BW25113 and *L. monocytogenes* were carried out as described in section Growth, Identification, and Characterization of the Environmental Strains.

### Data analysis

Plots and statistical analyses were carried out using the GraphPad Prism 6.0 (GraphPad Software, Inc.). Analysis of variance (ANOVA) and *t-*test were used considering *p* < 0.05. The statistical significance was indicated as follows: ^*^, for values where *p* < 0.05, ^**^, for values where *p* < 0.01, ^***^, for values where *p* < 0.001 and ^****^, for values where *p* < 0.000; ns, no significant difference.

## Results

### Isolation, identification, and characterization of metal(loid) resistant bacteria

A total of 47 bacterial strains isolated from environmental samples were assessed for metal(loid) resistance. Cells were routinely grown in LB or LB-agar plates at 37°C and exposed to different concentrations of 19 different, defined metal(loid)s. None of them grew in Cd^2+^-, Al^3+^-, or Mn^2+^-containing LB medium nor in R2A, Acidiphilium, or ATB culture media. Table S1 shows the ability to grow in the presence of the indicated metal(loid)s; when reduced, some of them (e.g., Te, Se, Au, and Ag) showed a characteristic color that was monitored spectrophotometrically. In turn, while strain MF47 was the most sensitive to all metal(loid)s tested, strains MF45 and MF46 tolerated only Pb^2+^ and Te^4+^, respectively.

**Figure 1:**
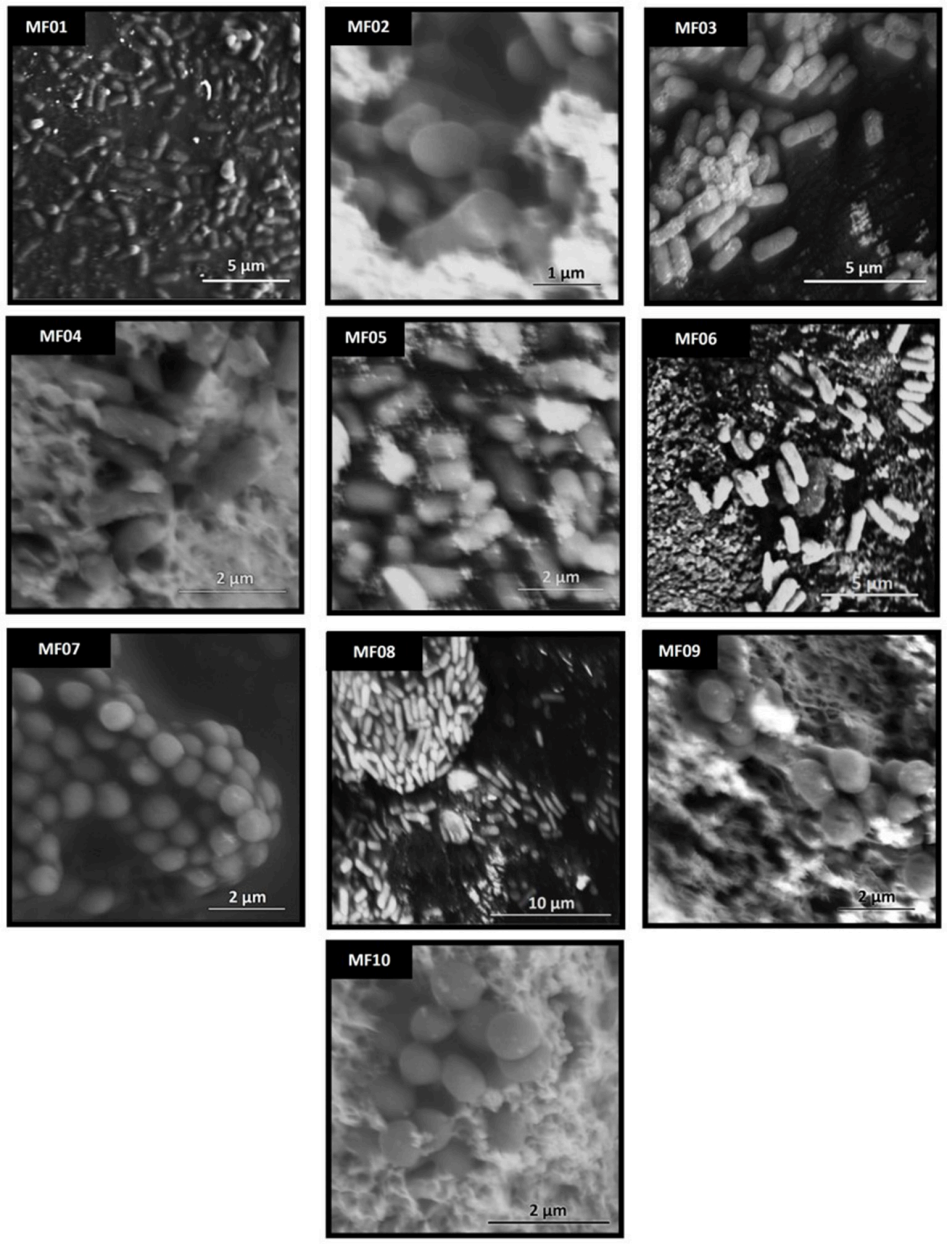
Scanning electron microscopy of the environmental multi-metal(loid) resistant bacterial strains. Cells were prepared for SEM as described in Methods. Each image represents a different strain (indicated in the black box).

Ten strains (MF01-MF10), including cocci, bacilli and short-bacilli, from which 21 and 26 were Gram negative and Gram positive, respectively (Table S2), were selected for further studies because of their ability to grow in the presence of 15, 12, 11, 11, 11, 9, 13, 9, 13, and 9 different metal(loid)s and/or to reduce them (Table S1). They belong to the *Enterobacter* (MF01 and MF04), *Staphylococcus* (MF02, MF07, and MF10), *Acinetobacter* (MF05 and MF09), and *Exiguobacterium* (MF03, MF06, and MF08) genera, as determined by 16S rRNA gene sequencing (Figure S1, Table S2). Their fine morphology was analyzed by scanning electron microscopy (Figure 1): MF02, MF07, and MF10 were coccoid, MF01, MF03, MF04, MF06, and MF08 showed bacillar forms and MF09 and MF05 were cocobacilli. Their physiological characteristics, as determined by biochemical tests, are listed in Table 1. Excepting MF03, all strains used citrate as carbon source, but none produced indole or hydrogen sulfide. All strains were oxidase negative and catalase positive and while MF05, MF06, MF07, MF08, MF09, and MF10 did not oxidize sugars at all, MF01 oxidized almost all sugars assayed but inositol.

**Table 1:** Physiological characterization of metal(loid)-resistant strains.

**Strain**	**1**	**2**	**3**	**4**	**5**	**6**	**7**	**8**	**9**	**10**
**UTILIZATION OF**										
Citrate	+	+	–	+	+	+	+	+	+	+
**PRODUCTION OF**										
H_2_S	–	–	–	–	–	–	–	–	–	–
Indole	–	–	–	–	–	–	–	–	–	–
Acetoin	–	–	+	–	+	+	+	+	+	+
NO_2_	+	+	–	+	–	+	–	+	–	–
N_2_	–	–	+	–	+	–	+	–	+	+
**TEST**										
Oxidase	–	–	–	–	–	–	–	–	–	–
Catalase	+	+	+	+	+	+	+	+	+	+
**PRESENCE OF**										
Arginine dihydrolase	+	–	–	+	–	+	+	+	+	+
Lysine decarboxylase	–	–	–	–	–	–	–	–	–	–
Ornithine decarboxylase	+	–	–	+	–	–	–	–	–	–
Urease	–	–	–	–	–	–	+	–	–	+
Tryptophane deaminase	–	–	–	–	–	–	–	–	–	–
Gelatinase	–	–	+	–	+	+	+	+	+	+
Alkaline phosphatase	+	+	+	+	–	+	–	+	–	–
Esterase (C4)	–	+	+	+	+	+	+	+	+	+
Esterase lipase (C8)	–	–	+	+	+	+	+	+	+	+
Lipase (C14)	–	–	–	–	–	–	–	–	–	–
Leucine arylamidase	+	–	–	+	+	+	–	–	+	–
Valine arylamidase	–	–	–	–	–	+	+	–	–	+
Cysteine arylamidase	–	–	–	+	+	–	–	–	+	–
Trypsin	–	–	–	–	–	–	+	–	–	–
α-chymotrypsin	–	–	–	–	–	+	–	–	–	–
Acid phosphatase	+	+	+	+	+	–	+	+	+	+
Naphthol-AS-BI-phosphohydrolase	+	+	+	+	+	+	+	+	+	+
α-galactosidase	–	–	–	–	–	–	–	–	–	–
β-galactosidase	+	–	–	+	–	–	–	–	–	–
β-glucuronidase	–	–	–	–	–	–	–	–	–	–
α-glucosidase	+	+	+	–	–	+	–	+	–	–
β-glucosidase	–	+	+	–	–	–	–	+	–	–
N-acetyl-β-glucosaminidase	–	–	–	–	–	–	–	–	–	–
α-mannosidase	–	–	–	–	–	–	–	–	–	–
α-fucosidase	–	–	–	–	–	–	–	–	–	–
**OXIDATION OF**										
D-glucose	+	+	–	+	–	–	–	–	–	–
D-mannitol	+	+	+	+	–	–	–	–	–	–
Inositol	–	–	–	–	–	–	–	–	–	–
D-sorbitol	+	–	–	+	–	–	–	–	–	–
L-rhamnose	+	–	–	–	–	–	–	–	–	–
D-saccharose	+	+	–	–	–	–	–	–	–	–
D-melibiose	+	–	–	–	–	–	–	–	–	–
Amygdaline	+	+	–	–	–	–	–	–	–	–
L-arabinose	+	–	–	–	–	–	–	–	–	–

*+, positive; −, negative. Strains: (1) MF01, (2) MF02, (3) MF03, (4) MF04, (5) MF05, (6) MF06, (7) MF07, (8) MF08, (9) MF09, and (10) MF10*.

### Metal(loid) resistance and reduction by the environmental strains

**Figure 2:**
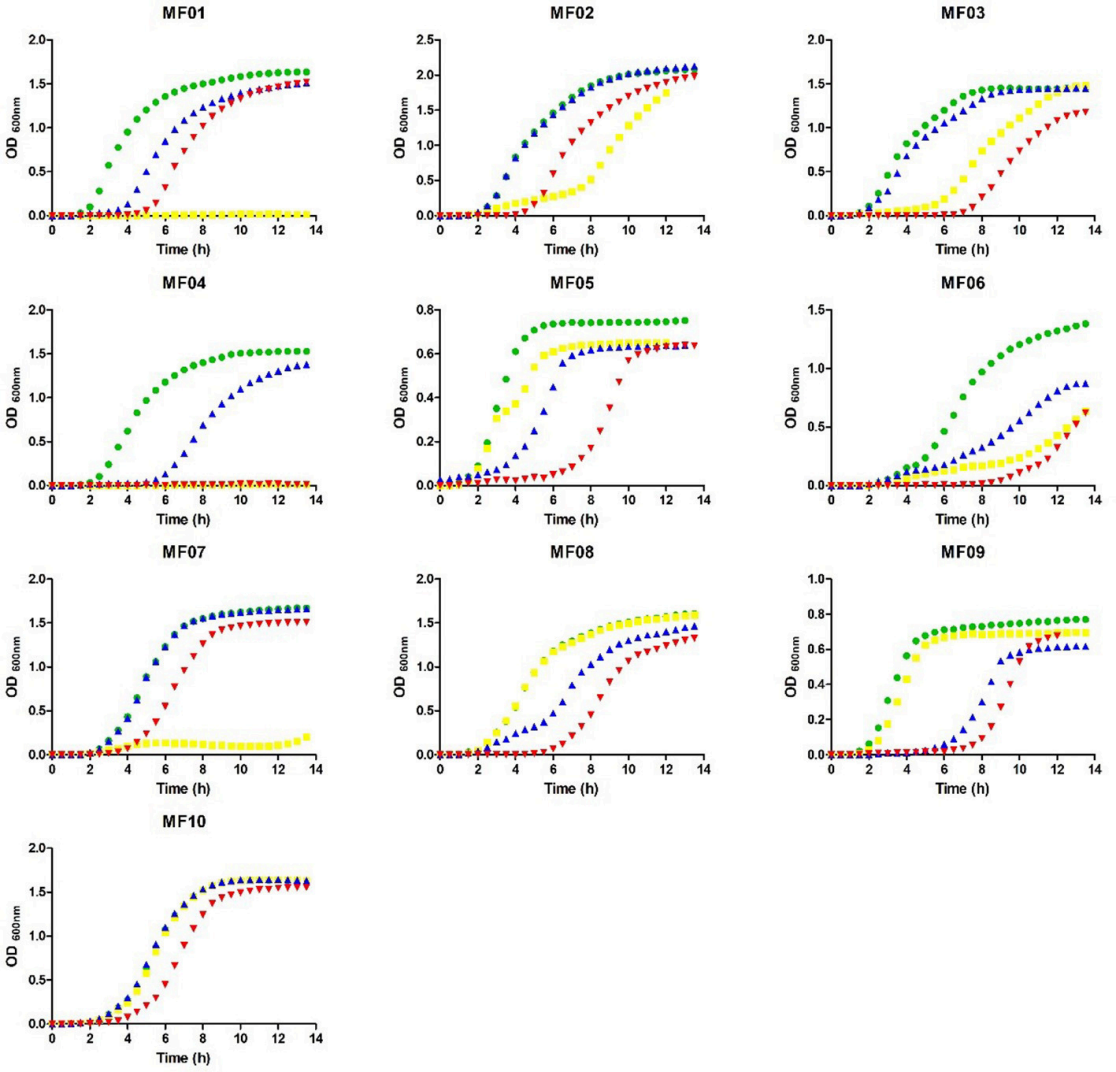
Growth curves of the environmental multi-metal(loid) resistant bacteria exposed to ${\text{TeO}}_{3}^{2-}$, ${\text{AuCl}}_{4}^{-}$, and Ag^+^. Cells were grown in LB medium in the absence (green) or presence of 4 μM ${\text{TeO}}_{3}^{2-}$ (yellow), 15 μM ${\text{AuCl}}_{4}^{-}$ (blue), or 15 μM Ag^+^ (red).

Minimal inhibitory concentrations of K_2_TeO_3_, AgNO_3_, HAuCl_4_, K_2_CrO_4_, CdCl_2_, NaAsO_2_, CuSO_4_, and Na_2_SeO_3_ were determined under aerobic growth conditions for the strains listed in Table S2. MF02 and MF04 were the most resistant and sensitive to tellurite, respectively. All strains exhibited similar resistance levels to HAuCl_4_, CdCl_2_, AgNO_3_, and CuSO_4_, while MF01 and MF03 were the most sensitive to NaAsO_2_. Differences up to 500-fold in the MIC of K_2_CrO_4_ were observed, MF02 and MF08 tolerating the highest concentration (400 mM). Finally, while MF01 and MF02 grew in the presence of Na_2_SeO_3_ up to 500 mM, MF10 was the most sensitive isolate (3.125 mM). Then, the effect of sublethal concentrations of ${\text{TeO}}_{3}^{2-}$, ${\text{AuCl}}_{4}^{-}$ and Ag^+^ on bacterial growth was assessed (Figure 2). Although an extended lag phase was observed, cells regained growth between ~4 and ~7 h after exposure to the metalloid. Growth of MF01, MF04, and MF07 was completely abolished in the presence of tellurite.

**Figure 3:**
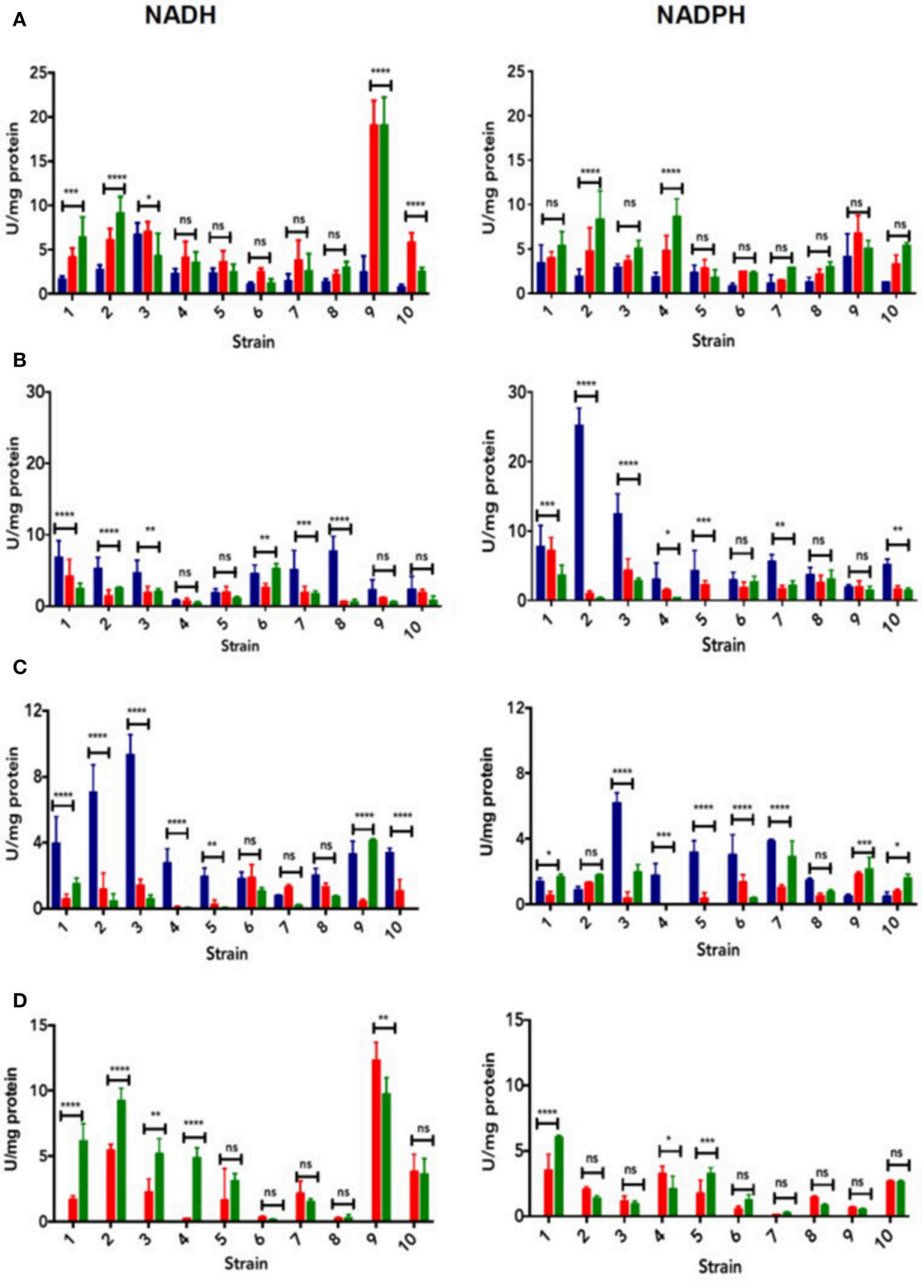
Metal(loid)-reducing activity. Reduction of tellurium (IV) **(A)**, selenium (IV) **(B)**, silver (I) **(C)**, and gold (III) **(D)**, in the presence of NADH (left) or NADPH (right). Colored bars indicate different pH values tested: 7.0 (blue), 8.0 (red), and 9.0 (green). Horizontal bars indicate the statistical analysis within the group. Data represent the average of 3 independent assays ± SD. Statistical significance was according to section Data analysis.

On the other hand, crude cell extracts were assayed to assess tellurium (IV)-, selenium (IV)-, gold (III)-, and silver (I)-reducing activity in the presence of NADH or NADPH and in the pH range 7–9 (Figure 3 and Figure S2). The reduction of three metal(loid)s generated a change of the solution's color: black, purple and yellow-orange in the case of tellurite (Figure S2A), ${\text{AuCl}}_{4}^{-}$ (Figure S2B) and Ag^+^ (Figure S2C), respectively. While MF09 was the best tellurium (IV) reducer (pH 8-9 with NADH as cofactor, Figure 3A), selenium (IV) was better reduced by MF02 at pH 7.0 using NADPH as cofactor (Figure 3B). In turn, MF03 exhibited the best Ag (I)-reducing activity at pH 7.0 in the presence of NADH (Figure 3C). Finally, a robust gold-reducing activity was observed in MF09 at pH 8.0 with NADH as the enzyme cofactor (Figure 3D).

To look for proteins which could be responsible of metal(loid) reduction, tellurite- and gold- reducing activities were assessed *in situ* by polyacrylamide gel electrophoresis under non-denaturing conditions (Figures S3A,B). Reduction bands generated by MF01 extracts were excised from the gel and fractionated by SDS-PAGE (Figure S3C). Twelve bands were identified by MALDI-TOF (Figure S3D) which included the enzyme glutathione reductase (GorA), belonging to the flavoprotein family. The ability of GorA from the Antarctic strain *Pseudomonas sp*. BNF22 to reduce ${\text{TeO}}_{3}^{2-}$ had been observed previously in our laboratory (Pugin et al., 2014). However, its ability to reduce other metal(loid)s and/or its efficiency has not yet been determined, which prompted us to focus on this phenomenon. The *gorA* gene was amplified from the *E. cloacae* genome and cloned into the pET/101 expression vector to generate the pET/*gorA* recombinant plasmid (Figures S3E,F). A kinetic induction assay showed a ~50 kDa protein band that accumulated over time (Figure S3G). The enzyme was purified and assayed for tellurium (IV), selenium (IV), gold (III), silver (I), and copper (II) reduction, measured in the presence of NADH or NADPH and in the pH range 6–9. GorA reduced optimally ${\text{TeO}}_{3}^{2-}$, ${\text{SeO}}_{3}^{2-}$ and Ag^+^ at pH 8.0, 6–7 and 7, respectively (Figure S3H).

### Synthesis and characterization of nanostructures

**Figure 4:**
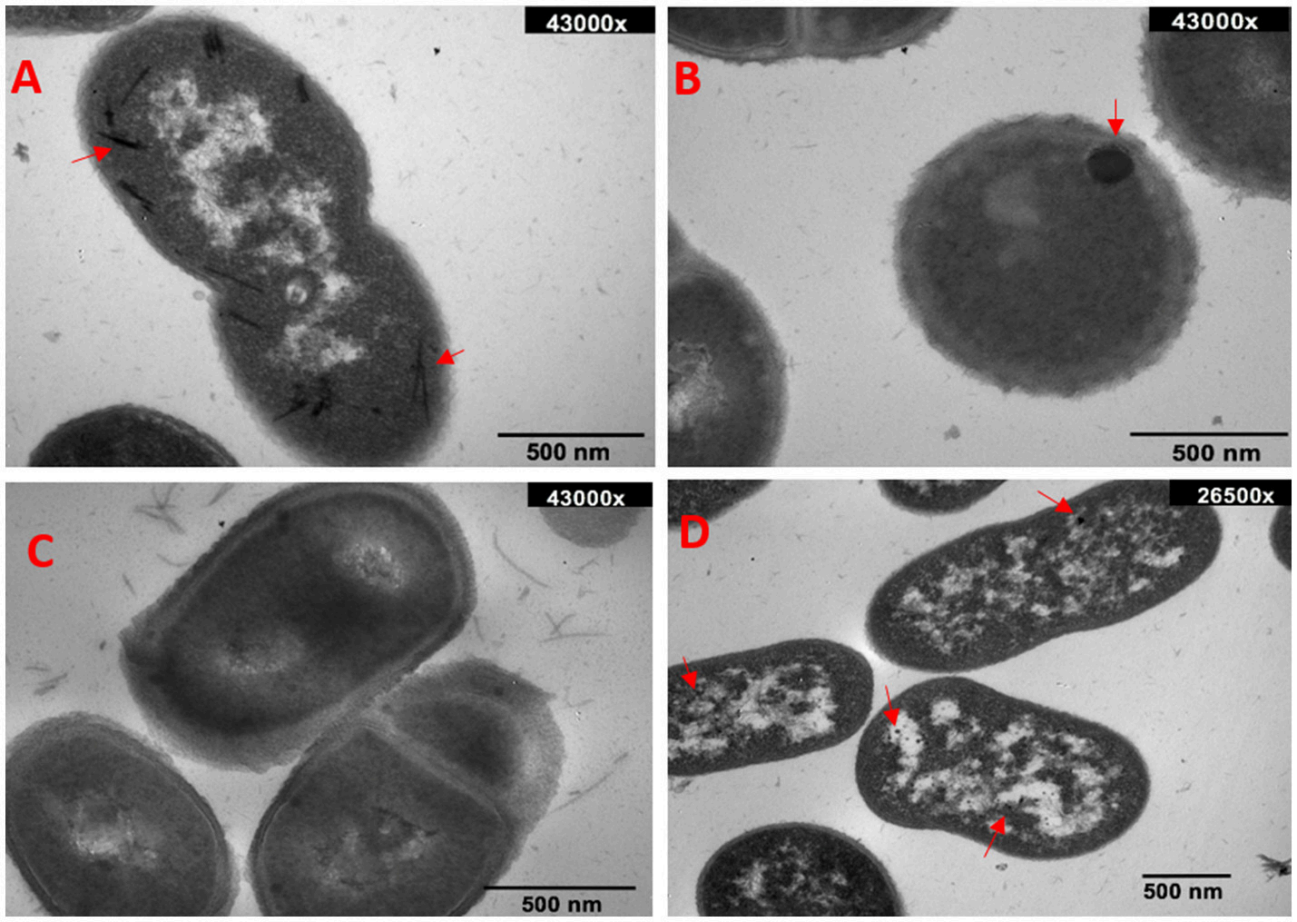
Electron micrographs of *in vivo* synthesized NS. TeNS, SeNS, AgNS, and AuNS synthesized *in vivo* by MF09 **(A)**, MF02 **(B)**, MF03 **(C)**, and MF09 **(D)**, respectively.

Ultrathin sections of selected strains that were exposed to sublethal concentrations (¼ of the MIC) of defined metal(loid)s were analyzed by transmission electron microscopy (Figure 4). Intracellular, evenly distributed long electron-dense rods, representing most probably tellurium nanostructures (TeNS) were observed in tellurite-exposed MF09 (Figure 4A). In turn, SeNS generated by MF02 appeared as spherical particles located near the cell membrane (Figure 4B). In both cases, alterations in size, morphology and cell division were observed (not shown). No silver-containing particles were generated by MF03 under the same conditions (Figure 4C). Finally, small, circular and evenly distributed gold particles were seen when MF09 was treated with sub lethal gold concentrations (Figure 4D).

**Figure 5:**
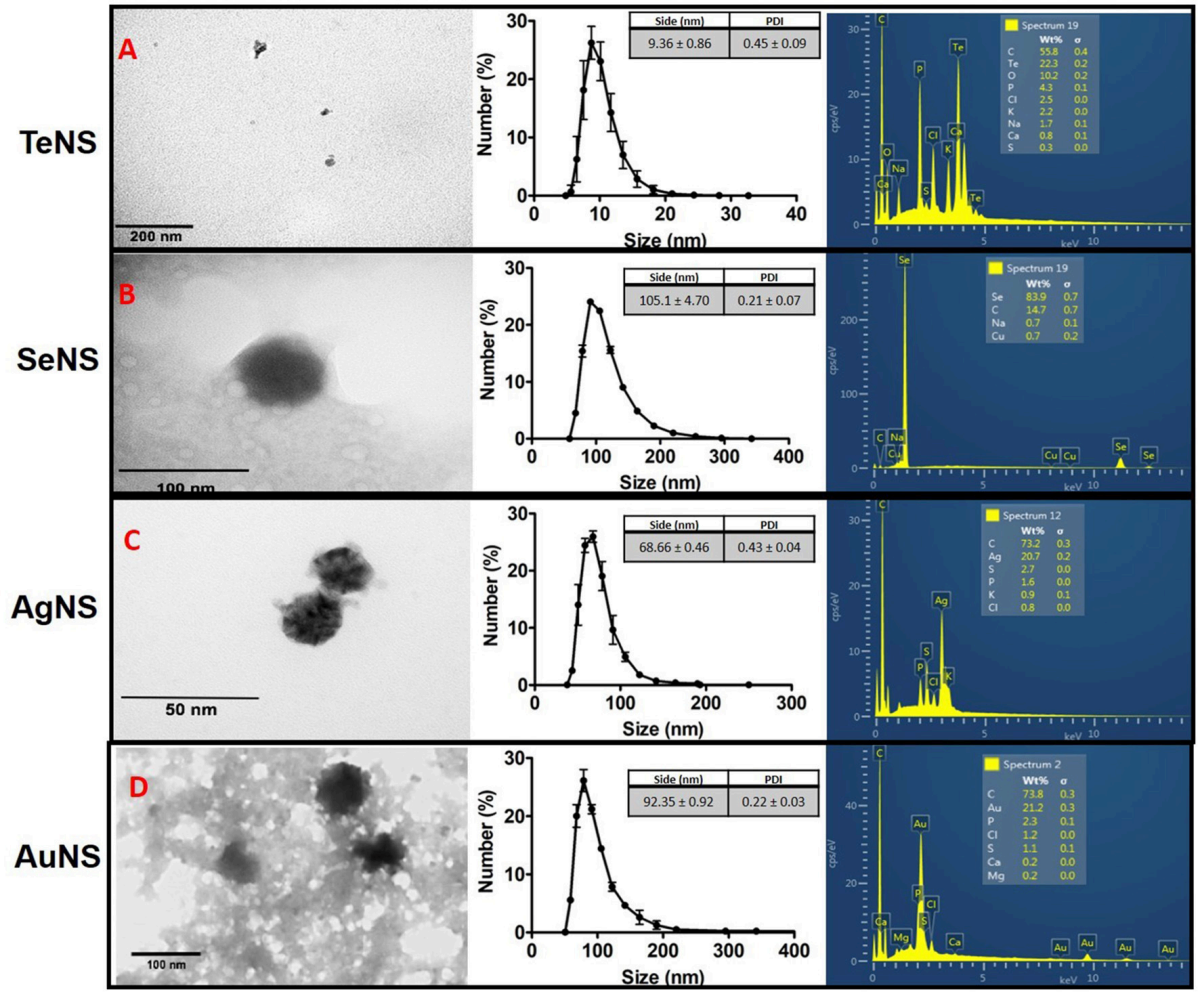
Characterization of *in vitro* synthesized NSs. Electron micrographs (left), dynamic light scattering (middle), and EDS (right) of NS synthetized by: MF09–TeNS– **(A)**; MF02–SeNS– **(B)**; MF03–AgNS– **(C)**; MF09–AuNS– **(D)**. Data represent the average of 3 independent trials ± SD.

On the other hand, *in vitro* generation of NS by bacterial crude extracts lasted ~16 h, irrespective of the strain or metal(loid) tested. NS morphology, size and chemical composition were determined by transmission electron microscopy (TEM), dynamic light scattering (DLS) and total reflection X-ray fluorescence spectrophotometry (TRXF), respectively. TeNS synthetized using MF09 extracts showed heterogeneous morphology with an average diameter ~10 nm (Figure 5A, left panel) and a hydrodynamic diameter ~9.36 nm (polydispersity index PDI 0.446), in agreement with TEM results (Figure 5A, middle panel). The chemical composition was 23.2% tellurium, 55.8% carbon and low amounts of chlorine, potassium, and phosphorus (Figure 5A, right panel). Moreover, TXRF analysis indicated that the tellurium concentration in TeNS was 78.66 ± 7.51 μg/ml. In the case of the SeNS generated with crude extracts from MF02, nanoparticles showed a spherical morphology with regular edges—nanospheres—and a size of ~100 nm (Figure 5B, middle panel); they showed a gaussian distribution (DLS) with a maximum at 105.1 nm (PDI 0.124) (Figure 5B, middle panel). Selenium content in SeNS was 83.9%, while TRXF showed that Se concentration was 158.98 ± 16,4 μg/ml (Figure 5B, right panel). On the other hand, AgNS generated using crude extracts from MF03 showed a size of ~10 nm, spherical-like shape with irregular edges and an electron-dense core (Figure 5C, left panel). These AgNS also showed a gaussian distribution with a maximum centered at 68.46 nm (PDI 0.43) (Figure 5B, middle panel), a size larger than that assessed by TEM; AgNS contained mostly carbon and to a lesser extent silver (9.84 ± 1.05 μg/ml, representing 20.7%) (Figure 5C, right panel). The same MF09 crude extract used to prepare TeNS was utilized to generate AuNS, whose morphology was not easy to determine because of the presence of organic material surrounding the particles (Figure 5D, left panel). Nevertheless, these NS were rather spherical with a size ~50 nm. AuNS showed a size distribution with a maximum at 82.35 nm (Figure 5D, middle panel). These nanoparticles contained 21.2% gold and other elements in lower amounts such as calcium, magnesium, chlorine, sulfur, and phosphorus (Figure 5A, right panel). TXRF analysis indicated that AuNS exhibited a gold concentration of 77.23 ± 12.61 μg/ml.

**Figure 6:**
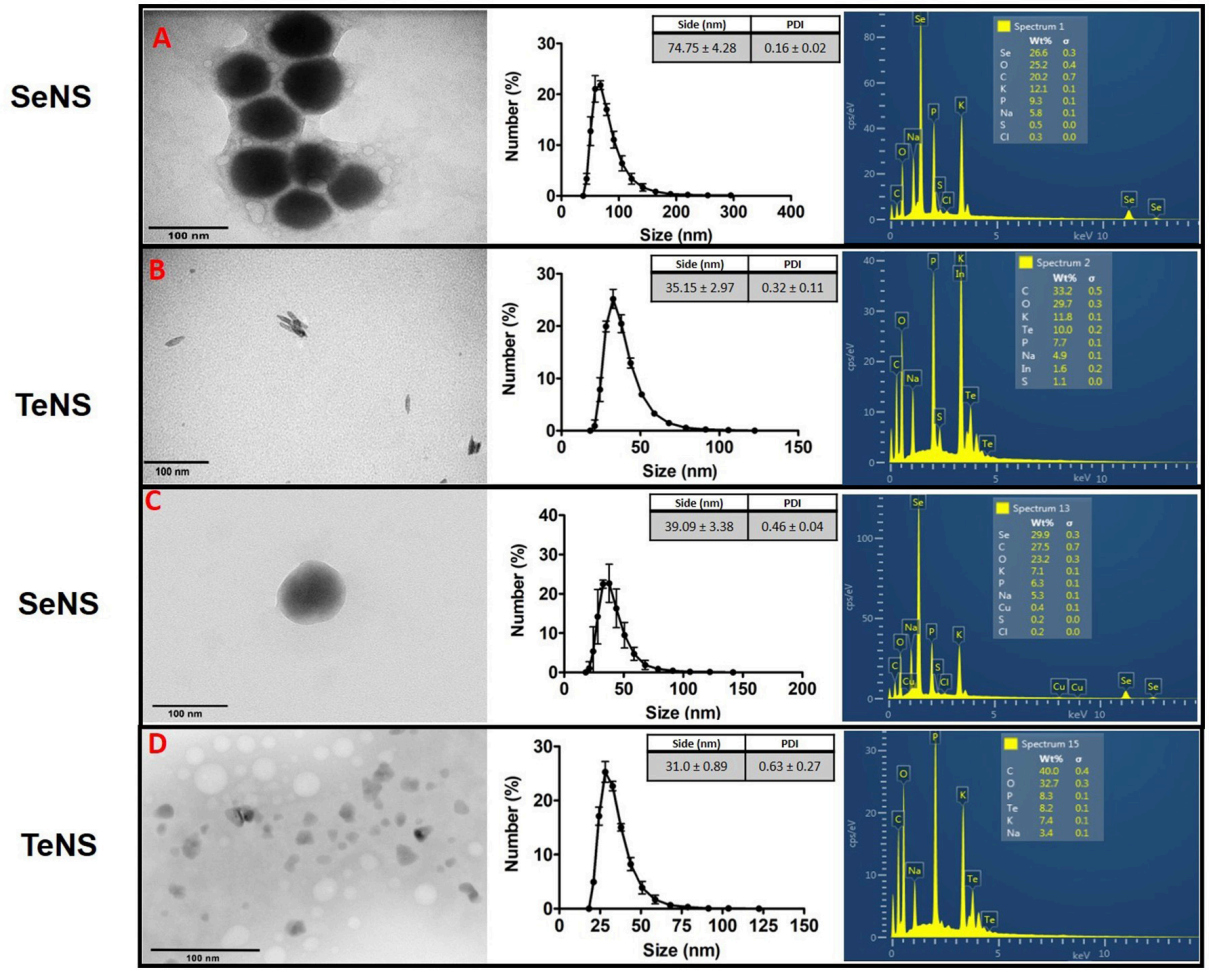
Characterization of GorA-synthesized NS. Electron micrographs (left), dynamic light scattering (middle), and EDS (right). Whereas the SeNS **(A)** and TeNS **(B)** were generated using purified GorA, SeNS **(C)** and TeNS **(D)** were generated using crude extracts of *E. coli* overexpressing the *gorA* gene. Numbers are the average of 3 independent trials ± SD.

Finally, crude extracts from GorA-overproducing *E. coli* as well as purified GorA were used to synthesize *in vitro* SeNS and TeNS (Figure 6): (i) both, crude extracts and purified protein were able to generate spherical SeNS with a size of ~50 nm and irregular edges (TEM). DLS analysis indicated that GorA-synthesized NS are prone to aggregation with a size distribution of 74.75 nm (PDI 0.159) (Figure 6A); conversely NS obtained using crude extracts did not aggregate and showed a size of 39.09 nm (PDI 0.456) (Figure 6C). SeNS from crude extracts showed a higher amount of selenium (131.65 ± 16.46 μg/ml, representing 29.9%) than GorA-SeNS (164.93 ± 24.7 μg/ml, representing 26.6%) (Figures 6A,C); (ii) in turn, GorA-TeNS showed a rather stick-like morphology (Figure 6B), while nanoparticles synthetized using crude extracts exhibited irregular, non-homogenous morphology, with some of them showing triangular shapes (Figure 6D). Size distribution (DLS) of GorA- and crude extract-TeNS was 35.11 nm (PDI 0.32) and 31 nm (PDI 0.63), respectively (Figures 6B,D). TeNS from crude extracts showed a higher amount of tellurium (127.36 ± 15.26 μg/ml, representing 10%) than GorA-TeNS (157.6 ± 25.3 μg/ml, representing 8.2%) (Figure 6B,D). Both crude extracts and purified protein showed the presence of other elements (more abundant in the GorA solution) such as potassium, sodium, sulfur, and phosphorus, among others.

### NS antibacterial activity

The minimal inhibitory concentration of *in vitro* synthesized NS was determined and along with the construction of growth curves, were used to assess their putative antibacterial effects. Aerobically synthesized TeNS using MF09 crude extracts showed MICs of 45- and 66- μg/ml for *E. coli* and *L. monocytogenes*, respectively. Similar MIC values (40 and 82 μg/ml, respectively) were observed for TeNS generated using GorA-overproducing *E. coli* crude extracts. In turn, AuNS MICs for *E. coli* and *L. monocytogenes* were 64- and 68- μg/ml, respectively. At the tested concentrations, both SeNS and AgNS did not inhibit *E. coli* or *L. monocytogenes* growth. However, Te- and Au-containing NS inhibited completely the growth of *E. coli* and *L. monocytogenes* (Figure S4).

## Discussion

Environmental bacteria survive in the presence of toxic agents such as metal(loid)s because they have evolved specific mechanism to cope with them (Summers and Silver, 1978; Bollag et al., 1994). Specifically, bacterial metal(loid)-reduction has gained attention among researchers since naturally reducing- or genetically modified- bacteria are widely used in the field of bioremediation (Stephen and Macnaughtont, 1999; Das et al., 2016) and biotechnology to generate nano-scaled structures exhibiting biomedical, electronic and pharmacological applications, among others (Suresh, 2012).

In this line, environmental bacteria from different Chilean regions were isolated, identified and characterized. Such regions exhibit a combination of many extreme factors like high salinity, desiccation, high and low temperatures, volcanic intervention, etc., most of them responsible for generating oxidative stress in organisms. Since metal(loid)s are also able to induce oxidative stress (Nies, 1999; Imlay, 2003), it was inferred that those places from where the samples were taken could host metal(loid)-resistant bacteria. The 10 most resistant to- and able of metal(loid)s reduction bacteria were chosen for further analyses because of their potential biotechnological applicability.

Their resistance level to 8 metal(loid)s was evaluated under aerobic conditions (Table S2). In this situation, most of these toxicants exert -at least in part- toxicity because of ROS production (mainly superoxide), causing the inactivation of certain enzymes and metabolic pathways, modification of nucleic acids and lipid peroxidation of the cell membrane (Ahearn et al., 1995; Calderón et al., 2009; Contreras and Vásquez, 2010; Lemire et al., 2013). High levels of tellurite resistance (4 mM) were observed for some isolates, while tellurite MIC did not exceed 0.004 mM for sensitive bacteria such as *E. coli* (Taylor, 1999). In the case of chromate and selenite, resistance levels observed for some isolates were up to ~500- (MF02 and MF08) and ~160-fold (MF01 and MF02) higher than that of other isolates, respectively. Interestingly, phylogenetically related strains (MF01 and MF04, MF09 and MF05, MF07 and MF10) exhibited different levels of resistance, probably because of the different environmental conditions where they thrive. Growth curves also showed that the isolates are differentially affected by these toxicants (Figure 3).

When reduced to their elemental state, tellurite, selenite, auric tetrachloride and silver generate colored, insoluble NS (Figure S2), which could serve as markers for putative biotechnological applications including medicine, electronics, pharmacy and engineering, among others (Ba et al., 2010; Suresh, 2012; Oves et al., 2013). Most strains exhibited metal(loid)-reducing ability (Figure 3, Figure S2), whose optimal reduction condition -pH range and enzyme cofactor among others- was determined. MF09 showed maximal tellurite reduction at pH 8 and 9 using NADH as electron donor (Figure 3A). This could be explained because, in general, redox centers of proteins exhibiting tellurite reductase activity contain reactive vicinal cysteines that are very sensitive to pH (Arenas et al., 2014b). Deprotonation of thiol groups of these cysteines generates the thiolate anion (-S^−^, pKa ~8.0) which is highly reactive (Vlamis-Gardikas, 2008). This high pKa implies that most cysteine thiols are protonated at pH 7.0 and hence, inactive.

Similar results were observed during ${\text{AuCl}}_{4}^{-}$ reduction by MF09 at pH 8.0 in the presence of NADH (Figure 3D). In turn, optimal selenite and silver reduction by MF03 and MF02, respectively, occurred at pH 7.0. Particularly, while Se^0^ is slowly oxidized to ${\text{SeO}}_{4}^{2-}$ in alkaline conditions (Kamal, 1994), ${\text{SeO}}_{3}^{2-}$ is reduced at more acidic pH values, thus favoring the formation elemental Se. On the other hand, pH influences metal(loid) speciation thus causing the formation of complexes and the deprotonation/protonation of functional groups that may participate in the catalytic process (Panda and Deepa, 2011). Also, pH can result in precipitation of defined ions (Atkins and Jones, 2006). For instance, Ag^+^ precipitates as silver hydroxide in alkaline conditions and is spontaneously transformed into the more stable, insoluble, silver oxide (Ag_2_O) (Burriel et al., 2006).

Next, the *in vivo* generation of tellurium-, selenium-, silver-, and gold-containing NS was evaluated using three of the environmental strains under study (Figure 4). TeNS generated by MF09 exhibited an elongated morphology, a situation that has been previously reported in *Rhodobacter capsulatus* (Turner et al., 2011). On the other hand, MF02-generated SeNS showed spherical morphology and a size >100 nm (Figure 4B). Actually, SeNS produced by most microorganisms exhibit spherical morphology and in some cases, transformation from spheres to nanowires do occur (Mane et al., 2015). In turn, MF03 did not form AgNS (Figure 4C), probably because Ag^+^ interacts with- and precipitate with a large number of intra- or extra-cellular anions (thus decreasing its availability to form NS) (Atkins and Jones, 2006); such is the case when ${\text{PO}}_{4}^{3-}$ present in the culture medium form stable compounds such as Ag_3_PO_4_ (Xiu et al., 2011). MF09- generated AuNS were observed as small intracellular deposits evenly distributed in the cell (Figure 4D). It general, AuNS synthesis is directly dependent on the microorganism and pH of the medium. In this line, marine yeasts form large-sized AuNPs at acidic pH values, while it reduced at alkaline pHs; conversely, fungi belonging to the *Verticillium* genus form larger AuNPs at alkaline pH values (Dhillon et al., 2012).

It should be noted that the molecular basis of the *in vivo* metal(loid)-reducing activity is very hard to determine accurately since a large number of factors that are present in the cell context may affect and/or influence this activity; this makes almost impossible to correlate directly *in vitro* activity and the generation of metal(loid) deposits *in vivo* and variables such as culture medium, toxicant exposure time, temperature, oxygen availability, among others, should be explored further.

The ability of crude extracts to generate metal(loid)-containing NS was used to characterize NS synthesis *in vitro*, including their morphology, size, composition and metal(loid)concentration (Figure 5). All the formed structures exhibited sizes <100 nm and hence, fall in the definition of nanostructures, i.e., at least one dimension does not exceed 100 nm (Suresh, 2012). MF09 crude extracts formed TeNS that were smaller in size than AuNS (Figures 5A,D). An important factor that affects NS morphology and size is related to the formation of the NS itself, where NS nucleation and growth are limiting steps for these NS characteristics (Xiong and Lu, 2015). Both TeNS and AuNS contain a high proportion of the respective metal(loid), 22.3 and 21.2%, respectively, in addition to other elements such as carbon. In particular, TeNS exhibited small sizes and poorly defined morphologies. Unfortunately, the presence of organic material hampered the correct visualization of AuNS. This did not affect SeNS observation, which showed a rather spherical morphology with poorly defined edges, and contained as much as 83.9% Se (Figure 5B). This result is consistent with the high Se concentration determined by TXRF (158.98 ± 16.4 μg/ml). Different was the case of AgNS, which contained lower metal abundance (Figure 5C) and is probably due to the presence of phosphate in the reaction buffer (see above).

Lastly, NS were also generated using purified GorA from *E. cloacae* (>92% pure) as well as crude extracts of a recombinant *E. coli* overexpressing the *E. cloacae gorA* gene (Figure 6). Given that it is a relatively new procedure, not much information is available in the literature. This flavoprotein, which was identified and characterized in this work, exhibits the ability to efficiently reduce ${\text{SeO}}_{3}^{2-}$ and ${\text{TeO}}_{3}^{2-}$ (Figure S3H). TeNS synthesized using crude extracts showed a rather triangular shape, while those generated by the purified protein were slightly larger size, irregular and exhibited a more spherical morphology (Figures 6B,D). As mentioned, difference in size and morphology could be attributed to the influence of the cellular environment on the metal(loid)-reducing efficiency.

Intracellular tellurite reduction could occur also because the participation of reductants such as gluthatione (GSH) and other cytosolic thiols (painter-type reactions) whose reducing ability would promote Te^0^ formation (Chasteen et al., 2009). Given that GorA is responsible for appropriate GSH levels, it is tempting to speculate that TeNS could be formed more efficiently when it is overproduced. This situation could explain why TeNS generated by crude extracts of GorA-overproducing *E. coli* are larger than those generated by the purified protein. No differences were observed regarding the Te content in TeNS. Actually, elemental analyses showed that Te was present in lower amount regarding other elements such as carbon, oxygen and phosphorus. Based on the stoichiometry of Te and O (oxygen percent is at least twice that of Te) in TeNS generated under aerobic conditions either by GorA or crude extracts, it could be inferred that in NS tellurium is present as tellurium dioxide (TeO_2_). Nevertheless, additional experimental evidence is required to state it definitely. It has been observed lately that GorA-generated TeNS remain attached to the enzyme, probably through covalent bonding with the active site catalytic cysteine residues (Pugin et al., 2014). Unlike TeNS, SeNS generated using crude extracts of GorA-overproducing cells were smaller than those produced by purified GorA (Figures 6A,C). This could be explained by some kind of electron leak, thus making selenite reduction less efficient (Zare et al., 2013; Mane et al., 2015). However, Se concentration was about the same in SeNS synthesized in both conditions.

Given that antibiotic-resistant, pathogenic bacteria have increased almost exponentially worldwide, the development of tools from biotechnological origin for biomedical applications is mandatory (Yacoby and Benhar, 2008). In this work, the antibacterial activity of the *in vitro*-generated NS was tested against *E. coli* and *L. monocytogenes*. TeNS made by crude extracts of GorA-overproducing *E. coli* were the most toxic for *E. coli* and *L. monocytogenes*. Similar results were observed with MF09-generated AuNS and TeNS (Figure S4). The effect of NS on *E. coli* and *L. monocytogenes* growth was also assessed by constructing growth curves. Results from Figure S4 show that in general all NS inhibited completely the bacterial growth.

Toxic effects of NS are generally related to the specific particle. Thus, AuNS have been widely used in therapeutic as well as diagnostic procedures, and exhibit antibacterial activity against *Salmonella* sp., *Bacillus subtilis, E. coli* and *Pseudomonas aeruginosa* (Das et al., 2009). In particular, chemically-generated AuNS seem to exert their toxicity in two ways: (i) changing membrane potential and inhibiting ATP synthase activity, which results in a general turn down of metabolism and (ii) inhibiting the binding of tRNAs to the ribosome, thus generating a global collapse of biological processes. Other NS are toxic because the release of metal ions, as in the case of some AgNS; in fact, Ag^+^ release causes damage to the cell membrane, inactivates proteins and inhibits DNA replication (Chaloupka et al., 2010; Xiu et al., 2012).

Our results show that in general all tested NS were more toxic for *E. coli* as compared to *L. monocytogenes*, which, as a Gram positive organism, possess a cell wall that probably retards or prevents the entry of NS. In this context, no information on the mechanism of NS uptake is available in the literature. Unlike TeNS and AuNS, *in vitro*-synthesized SeNS and AgNS did not inhibit bacterial growth (not shown). Results with SeNS could be explained because selenium is an essential element for microorganisms (Lemire et al., 2013), and probably higher Se amounts in NS would be needed to generate toxicity. It has been also shown that SeNS inhibits both growth and proliferation of cancer cells in culture (Selenius et al., 2010). On the other hand and although AgNS have been reported as the most toxic NS (Rai et al., 2009; Lin et al., 2012), they did not exhibit toxicity for the tested bacteria.

Finally, it seems very interesting that the same enzyme can reduce two metalloids possessing different chemical characteristics, as for instance, their reactivity with certain enzymes (Rigobello et al., 2011). We trust that the results of this work will help to define the molecular basis governing the biological synthesis of many potentially useful nanostructures.

## Conclusion

Ten bacteria exhibiting resistance to various metals and metalloids were isolated and characterized from environmental samples. Whole cells as well as crude extracts derived from them were successfully used for the biological synthesis of TeNS, SeNS, AuNS, and AgNS, which exhibited different, specific characteristics. In particular, *E. cloacae* was used as model to look for toxicant-reducing enzymes. In this context, it was found that purified *E. cloacae* GorA reduced efficiently ${\text{TeO}}_{3}^{2-}$ and ${\text{SeO}}_{3}^{2-}$. Finally, especially interesting were TeNS and AuNS, which exhibited antibacterial properties and inhibited *E. coli* and *L. monocytogenes* growth.

## Author contributions

MF, VF, MA-S, CM-V, FC, EM, DA, CV, and FA: Conceived and designed the experiments; MF, VF, EV, ML, DA, FC, MA-S, and CM-V: Performed the experiments; MF, VF, DA, MA-S, EM, ML, EV CM-V, CV, and FA: Analyzed the data; MA-S, CV, ML, and FA: Contributed reagents, materials, analysis tools; CV and FA: Wrote the paper.

### Conflict of interest statement

The authors declare that the research was conducted in the absence of any commercial or financial relationships that could be construed as a potential conflict of interest.

## Supplementary material

Figure S1 Phylogenetic trees based on the 16S rRNA gene sequence. Trees were constructed using the *neighbor-joining* method as described in Methods. **(A)** *Exiguobacterium*, **(B)** *Enterobacter*, **(C)** *Acinetobacter*, and **(D)** *Staphylococcus*. The numbers on the nodes represent the percentage of bootstrap (1,000 repetitions).

Figure S2 *in vitro* metal(loid)- reduction by crude extracts of the indicated strains. The reduction of ${\text{TeO}}_{3}^{2-}$**(A)**, ${\text{AuCl}}_{4}^{-}$**(B)**, and Ag^+^**(C)** was performed as described in Methods. The activity was measured at different pH values and monitored for 24 h. Strains analyzed: (1) MF01, (2) MF02, (3) MF03, (4) MF04, (5) MF05, (6) MF06, (7) MF07, (8) MF08, (9) MF09, (10) MF10.

Figure S3 Identification, cloning, and purification of GorA. Tellurite **(A)** and gold **(B)** reductase activity *in situ*. Crude extracts from each strain [MF01 (1), MF02 (2), MF03 (3), MF04 (4), MF05 (5), MF06 (6), MF07 (7), MF08 (8), MF09 (9), MF10(10)] were fractionated by NATIVE-PAGE and revealed as ${\text{TeO}}_{3}^{2-}$ or ${\text{AuCl}}_{4}^{-}$ activity as described in Methods. **(C)** SDS-PAGE of the reduction bands of ${\text{TeO}}_{3}^{2-}$ and ${\text{AuCl}}_{4}^{-}$ from MF01 extracts; Lanes 1, 2, and 5: molecular weight standards (BioRad, #1610305). Lanes 3 and 4: fractionation of proteins present in the reduction band of ${\text{TeO}}_{3}^{2-}$ and ${\text{AuCl}}_{4}^{-}$, respectively. **(D)** Identification of protein bands by MALDI-TOF. **(E)** Agarose gel electrophoresis of PCR-amplified *gorA*; Lane 1, molecular size standards (1 kb DNA ladder, Promega). Lane 2, amplification of *gorA*. **(F)** Agarose gel electrophoresis of *gorA* amplification by PCR. Lane 1, molecular size standards (1 kb DNA ladder, Invitrogen); lane 2, colony PCR amplification of *gorA*; lane 3, directional cloning of *gorA* assessed by PCR using the primers Gforward and pETReverse described in section Growth, identification and characterization of the environmental strains; **(G)** Induction kinetics and purification of GorA*;* SDS-PAGE of crude extracts of *E. coli* overexpressing *gorA* and the purified enzyme; ST, SDS-PAGE molecular weight standards (low range). Lanes 1-6, induction kinetics for GorA at 0, 1, 2, 3, 4, and 16 h, respectively. Lane 7, purified recombinant GorA. **(H)** GorA metal(loid)-reductase activity (U/mg protein), showing the dependence on the pH and cofactor.

Figure S4 Growth curves of *E. coli* and *L. monocytogenes* exposed to NS. Effect of TeNS synthesized by crude extracts of cells overexpressing the *E. cloacae gorA* gene in *E. coli* **(A)** and *L. monocytogenes* **(B)**. Effect of TeNS synthesized using crude extracts of MF09 in *E. coli* **(C)** and *L. monocytogenes* **(D)**. Effect of AuNS synthesized by crude extracts of MF09 in *E. coli* **(E)** and *L. monocytogenes* **(F)**. Black and blue lines represent the growth of control and NS-treated cells, respectively.

Table S1 Metal(loid)-resistant strains.

Table S2 MICs for the metal(loid)-resistant strains.

## References

[B1] Ahearn D. G. May M. M. Gabriel M. M. (1995). Adherence of organisms to silver-coated surfaces. J. Ind. Microbiol. 15, 372–376. 10.1007/BF01569993 8605074

[B2] Amstad E. Textor M. Reimhult E. (2011). Stabilization and functionalization of iron oxide nanoparticles for biomedical applications. Nanoscale 3, 2819–2843. 10.1039/c1nr10173k 21629911

[B3] Arenas F. A. Leal C. A. Pinto C. A. Arenas-Salinas M. A. Morales W. A. Cornejo F. A.. (2014b). On the mechanism underlying tellurite reduction by *Aeromonas caviae* ST dihydrolipoamide dehydrogenase. Biochimie 102, 174–182. 10.1016/j.biochi.2014.03.008 24680738

[B4] Arenas F. A. Pugin B. Henríquez N. A. Arenas-Salinas M. A. Díaz-Vásquez W. A. Pozo M. F. (2014a). Isolation, identification and characterization of tellurite hyper-resistant, tellurite-reducing bacteria from Antarctica. Polar Sci. 8, 40–52. 10.1016/j.polar.2014.01.001

[B5] Arenas-Salinas M. Vargas J. I. Morales W. Pinto C. Muñoz P. Cornejo F. A.. (2016). Flavoprotein-mediated tellurite reduction: structural basis and applications to the synthesis of tellurium-containing nanostructures. Front. Microbiol. 7:1160. 10.3389/fmicb.2016.01160 27507969 PMC4960239

[B6] Atkins P. Jones L. (2006). Principles of Chemistry. The Ways of Discovery. Madrid: Panamericana.

[B7] Azam A. Ahmed A. S. Oves M. Khan M. S. Habib S. S. Memic A. (2012). Antimicrobial activity of metal oxide nanoparticles against Gram-positive and Gram-negative bacteria: a comparative study. Int. J. Nanomed. 7, 6003–6009. 10.2147/IJN.S35347 23233805 PMC3519005

[B8] Ba L. A. Döring M. Jamier J. Jacob C. (2010). Tellurite: an element with great biological potency and potential. Org. Biomol. Chem. 8, 4203–4216. 10.1039/c0ob00086h 20714663

[B9] Bollag J. M. Mertz T. Otjen L. (1994). Role of microorganisms in soil bioremediation, in Bioremediation Through Rhizosphere Technology, ACS Symposium Series, eds Anderson T. Coats J. (Washington, DC: American Chemical Society), 2–10.

[B10] Borsetti F. Tremaroli V. Michelacci F. Borghese R. Winterstein C. Dalda F.. (2005). Tellurite effects on *Rhodobacter capsulatus* cell viability and superoxide dismutase activity under oxidative stress conditions. Res. Microbiol. 156, 7–13. 10.1016/j.resmic.2005.03.011 15946826

[B11] Burriel F. Lucena F. Arribas S. Hernández F. (2006). Analytical chemistry of the cations: Silver, in Qualitative Analytical Chemistry, ed Carmen de la Fuente (Madrid: Thomson), 419–426.

[B12] Calderón I. L. Elías A. O. Fuentes E. L. Pradenas G. A. Castro M. E. Arenas F. A. (2009). Tellurite-mediated disabling of [4Fe-4S] clusters of *Escherichia coli* dehydratases. Microbiology 155, 40–46. 10.1099/mic.0.026260-0 19383690

[B13] Carotti S. Marcon M. Marussich M. Mazzei T. Messori L. Mini E.. (2000). Cytotoxicity and DNA binding properties of a chloro glycythistidinate gold (III) complex (GHAu). Chem. Biol. Interact. 125, 29–38. 10.1016/S0009-2797(99)00160-X 10724364

[B14] Chaloupka K. Malam Y. Seifalian A. M. (2010). Nanosilver as a new generation of nanoproduct in biomedical applications. Trends Biotechnol. 28, 580–588. 10.1016/j.tibtech.2010.07.006 20724010

[B15] Chasteen T. G. Fuentes D. E. Tantaleán J. C. Vásquez C. C. (2009). Tellurite: history, oxidative stress, and molecular mechanisms of resistance. FEMS Microbiol. Rev. 33, 1–13. 10.1111/j.1574-6976.2009.00177.x 19368559

[B16] Cioffi N. Rai M. (eds). (2012). Nano-Antimicrobials: Progress and Prospects. Berlin: Springer Science & Business Media.

[B17] Contreras N. P. Vásquez C. C. (2010). Tellurite-induced carbonylation of the *Escherichia coli* pyruvate dehydrogenase multienzyme complex. Arch. Microbiol. 192, 969–973. 10.1007/s00203-010-0624-2 20821193

[B18] Correa-Llantén D. Muñoz-Ibacache S. Castro M. Muñoz P. Blamey J. (2013). Gold nanoparticles synthesized by *Geobacillus* sp. strain ID17 a thermophilic bacterium isolated from deception Island, Antarctica. Microb. Cell Fact. 12:75. 10.1186/1475-2859-12-75 23919572 PMC3751291

[B19] Cui Y. Zhao Y. Tian Y. Zhang W. Lu X. Jiang X. (2012). The molecular mechanism of action of bacterial gold nanoparticles on *E. coli*. Biomaterials 33, 2327–2333. 10.1016/j.biomaterials.2011.11.057 22182745

[B20] Das S. Dash H. Chakraborty J. (2016). Genetic basis and importance of metal resistant genes in bacteria for bioremediation of contaminated environments with toxic metal pollutants. Appl. Microbiol. Biotechnol. 100, 2967–2984. 10.1007/s00253-016-7364-4 26860944

[B21] Das S. K. Das A. R. Guha A. K. (2009). Gold nanoparticles: microbial synthesis and application in water hygiene management. Langmuir 25, 8192–8199. 10.1021/la900585p 19425601

[B22] Dhillon G. S. Brar S. K. Kaur S. Verma M. (2012). Green approach for nanoparticle biosynthesis by fungi: current trends and applications. Crit. Rev. Biotechnol. 32, 49–73. 10.3109/07388551.2010.550568 21696293

[B23] Dizaj S. M. Lotfipour F. Barzegar-Jalali M. Zarrintan M. H. Adibkia H. (2014). Antimicrobial activity of the metals and metal oxides nanoparticles. Mater. Sci. Eng. C 44, 278–284. 10.1016/j.msec.2014.08.031 25280707

[B24] Gurunathan S. Kalishwaralal K. Vaidyanathan R. Deepak V. Pandian S. Muniyandi J. (2009). Biosynthesis, purification and characterization of silver nanoparticles using *Escherichia coli*. Colloids Surf. 50, 328–335. 10.1016/j.colsurfb.2009.07.048 19716685

[B25] Imlay J. A. (2003). Pathways of oxidative damage. Annu. Rev. Microbiol. 57, 395–418. 10.1146/annurev.micro.57.030502.090938 14527285

[B26] Kamal M. A. (1994). Sodium selenate and sodium selenite. Natl. Toxicol. Progr. 48, 2–70.

[B27] Klonowska A. Heulin T. Vermeglio A. (2005). Selenite and tellurite reduction by *Shewanella oneidensis*. Appl. Environ. Microbiol. 71, 5607–5609. 10.1128/AEM.71.9.5607-5609.2005 16151159 PMC1214622

[B28] Kumar S. A. Abyaneh M. K. Gosavl S. W. Kulkarni S. K. Pasricha R. Ahmad A. (2007). Nitrate reductase-mediated synthesis of silver nanoparticles from AgNO_3_. Biotechnol. Lett. 29, 439–445. 10.1007/s10529-006-9256-7 17237973

[B29] Lemire J. A. Harrison J. J. Turner R. J. (2013). Antimicrobial activity of metals: mechanisms, molecular targets and applications. Nat. Rev. Microbiol. 11, 371–384. 10.1038/nrmicro3028 23669886

[B30] Li W. R. Xie X. B. Shi Q. S. Duan S. S. Ouyang Y. S. Chen Y. B. (2011). Antibacterial effect of silver nanoparticles on *Staphylococcus aureus*. Biometals 24, 135–141. 10.1007/s10534-010-9381-6 20938718

[B31] Lin Z. N. Lee C. H. Chang H. Y. Chang H. T. (2012). Antibacterial activities of tellurium nanomaterials. Chem. Asian J. 7, 930–934. 10.1002/asia.201101006 22438287

[B32] Mane R. S. Naushad M. Shirsat S. Kadam A. S. (2015). Selenium nanostructures: microbial synthesis and applications. RSC Adv. 112, 1–9. 10.1039/C5RA17921A

[B33] Narayanan K. B. Sakthivel N. (2010). Biological synthesis of metal nanoparticles by microbes. Adv. Colloid Interface Sci. 156, 1–13. 10.1016/j.cis.2010.02.001 20181326

[B34] Nies D. H. (1999). Microbial heavy-metal resistance. Appl. Microbiol. Biotechnol. 51, 730–750. 10.1007/s002530051457 10422221

[B35] Nies D. H. Grass G. (2009). Transition metal homeostasis. EcoSal Plus 3, 730–750. 10.1128/ecosalplus.5.4.4.3 26443772

[B36] Nies D. H. Silver S. (1995). Ion efflux systems involved in bacterial metal resistance. J. Ind. Microbiol. 14, 186–199. 10.1007/BF01569902 7766211

[B37] Oves M. Khan M. S. Zaidi A. Ahmed A. S. Ahmed F. Ahmad E.. (2013). Antibacterial and cytotoxic efficacy of extracellular silver nanoparticles biofabricated from chromium reducing novel OS4 strain of Stenotrophomonas maltophilia. PLoS ONE 8:e59140. 10.1371/journal.pone.0059140 23555625 PMC3605433

[B38] Oves M. Qari H. A. Felemban N. M. Khan M. Z. Rehan Z. A. Ismail I. M. I. (2017). Marinobacter lipolyticus from Red Sea for lipase production and modulation of silver nanomaterials for anti-candidal activities. IET Nanobiotechnol. 11, 403–410. 10.1049/iet-nbt.2016.0104 28530189 PMC8676228

[B39] Panda T. Deepa K. (2011). Biosynthesis of gold nanoparticles. J. Nanosci. Nanotechnol. 11, 10279–10294. 10.1166/jnn.2011.5021 22408900

[B40] Pérez J. M. Calderón I. L. Arenas F. A. Fuentes D. E. Pradenas G. A. Fuentes E. L.. (2007). Bacterial toxicity of potassium tellurite: unveiling an ancient enigma. PLoS ONE 2:e211. 10.1371/journal.pone.0000211 17299591 PMC1784070

[B41] Pradenas G. A. Díaz-Vásquez W. A. Pérez-Donoso J. M. Vásquez C. C. (2013). Monounsaturated fatty acids are substrates for aldehyde generation in tellurite-exposed *Escherichia coli*. Biomed Res. Int. 2013:563756. 10.1155/2013/563756 23991420 PMC3749545

[B42] Pugin B. Cornejo F. Muñoz P. Muñoz C. Vargas J. Arenas F.. (2014). Glutathione reductase-mediated synthesis of tellurium-containing nanostructures exhibiting antibacterial properties. Appl. Environ. Microbiol. 80, 7061–7070. 10.1128/AEM.02207-14 25193000 PMC4249020

[B43] Rai M. Yadav A. Gade A. (2009). Silver nanoparticles as a new generation of antimicrobials. Biotechnol. Adv. 27, 76–83. 10.1016/j.biotechadv.2008.09.002 18854209

[B44] Rao J. (2008). Shedding light on tumors using nanoparticles. ACS Nano 2, 1984–1986. 10.1021/nn800669n 19206441

[B45] Rigobello M. Folda A. Citta A. Scutari G. Gandin V. Fernandes A.. (2011). Interaction of selenite and tellurite with thiol-dependent redox enzymes: kinetics and mitochondrial implications. Free Radical Biol. Med. 50, 1620–1629. 10.1016/j.freeradbiomed.2011.03.006 21397686

[B46] Saitou N. Nei M. (1987). The neighbor-joining method: a new method for reconstructing phylogenetic trees. Mol. Biol. Evol. 4, 406–425. 3447015 10.1093/oxfordjournals.molbev.a040454

[B47] Sambrook J. Russell D. (2001). Molecular Cloning. A Laboratory Manual, 3rd Edn. Cold Spring Harbor, NY: Cold Spring Harbor Laboratory Press.

[B48] Selenius M. Rundlöf A. Olm E. Fernandes A. Björnstedt M. (2010). Selenium and the selenoprotein thioredoxin reductase in the prevention, treatment and diagnostics of cancer. Antioxid. Redox Signal. 12, 867–880. 10.1089/ars.2009.2884 19769465

[B49] Sharma V. K. Yngard R. A. Lin Y. (2009). Silver nanoparticles: green synthesis and their antimicrobial activities. Adv. Colloid Interface Sci. 145, 83–96. 10.1016/j.cis.2008.09.002 18945421

[B50] Silver S. Phung T. (2005). A bacterial view of the periodic table: genes and proteins for toxic ions. J. Ind. Microbiol. Biotechnol. 32, 587–605. 10.1007/s10295-005-0019-6 16133099

[B51] Song D. Li X. Cheng Y. Xiao X. Lu Z. Wang Y.. (2017). Aerobic biogenesis of selenium nanoparticles by *Enterobacter cloacae* Z0206 as a consequence of fumarate reductase mediated selenite reduction. Sci. Rep. 7, 1–10. 10.1038/s41598-017-03558-3 28607388 PMC5468319

[B52] Stephen J. R. Macnaughtont S. J. (1999). Developments in terrestrial bacterial remediation of metals. Curr. Opin. Biotechnol. 10, 230–233. 10.1016/S0958-1669(99)80040-8 10361068

[B53] Stolz J. F. Oremland R. S. (1999). Bacterial respiration of arsenic and selenium. FEMS Microbiol. Rev. 23, 615–627. 10.1111/j.1574-6976.1999.tb00416.x 10525169

[B54] Summers A. Silver S. (1978). Microbial transformations of metals. Annu. Rev. Microbiol. 32, 637–672. 10.1146/annurev.mi.32.100178.003225 360977

[B55] Suresh A. K. (2012). Metallic Nanocrystallites and Their Interaction with Microbial Systems. Springer Briefs in Molecular Science Biometals. Dordrecht; Heidelberg; New York, NY; London: Springer.

[B56] Tamura K. Nei M. Kumar S. (2004). Prospects for inferring very large phylogenies by using the neighbor-joining method. Proc. Natl. Acad. Sci. U.S.A. 101, 11030–11035. 10.1073/pnas.0404206101 15258291 PMC491989

[B57] Taylor D. E. (1999). Bacterial tellurite resistance. Trends Microbiol. 7, 111–115. 10.1016/S0966-842X(99)01454-7 10203839

[B58] Thakkar K. Mhatre S. Parikh R. (2010). Biological synthesis of metallic nanoparticles. Nanomedicine 23, 257–262. 10.1016/j.nano.2009.07.002 19616126

[B59] Tremaroli V. Fedi S. Zannoni D. (2007). Evidence for a tellurite-dependent generation of reactive oxygen species and absence of a tellurite-mediated adaptive response to oxidative stress in cells of *Pseudomonas pseudoalcaligenes* KF707. Arch. Microbiol. 187, 127–135. 10.1007/s00203-006-0179-4 17013634

[B60] Turner R. J. Borghese R. Zannoni D. (2011). Microbial reduction of tellurium metalloids as a tool in biotechnology. Biotechnol. Adv. 30, 954–963. 10.1016/j.biotechadv.2011.08.018 21907273

[B61] Vlamis-Gardikas A. (2008). The multiple functions of the thiol-based electron flow pathways of *Escherichia coli*: eternal concepts revisited. Biochim. Biophys. Acta 1780, 1170–1200. 10.1016/j.bbagen.2008.03.013 18423382

[B62] Vrionis H. A. Wang S. Haslam B. Turner R. J. (2015). Selenite protection of tellurite toxicity toward *Escherichia coli*. Front. Mol. Biosci. 69, 1–10. 10.3389/fmolb.2015.00069 PMC4683179 26732755

[B63] Xiong Y. Lu X. (2015). Metallic Nanoestructures: From Controlled Synthesis to Applications. Cham: Springer International publishing.

[B64] Xiu Z. M. Ma J. Alvarez P. J. (2011). Differential effect of common ligands and molecular oxygen on antimicrobial activity of silver nanoparticles versus silver ions. Environ. Sci. Technol. 45, 9003–9008. 10.1021/es201918f 21950450

[B65] Xiu Z. M. Zhang Q. B. Puppala H. L. Colvin V. L. Alvarez P. J. (2012). Negligible particle-specific antibacterial activity of silver nanoparticles. Nano Lett. 12, 4271–4275. 10.1021/nl301934w 22765771

[B66] Yacoby I. Benhar I. (2008). Antibacterial nanomedicine. Nanomedicine 3, 329–341. 10.2217/17435889.3.3.329 18510428

[B67] Zannoni D. Borsetti F. Harrison J. J. Turner R. J. (2008). The bacterial response to the chalcogen metalloids Se and Te. Adv. Microb. Ecol. 53, 1–72. 10.1016/S0065-2911(07)53001-8 17707143

[B68] Zare B. Babaie S. Setayesh N. Shahverdi A. R. (2013). Isolation and characterization of a fungus for extracellular synthesis of small selenium nanoparticles. Nanomedicine 1, 13–19. 10.7508/nmj.2013.01.002

