# Supplementary figures and images for: Synthesis and Antibacterial Activity of Metal(loid) Nanostructures by Environmental Multi-Metal(loid) Resistant Bacteria and Metal(loid)-Reducing Flavoproteins
Source: Front Microbiol. 2018 May 15;9:959. doi: 10.3389/fmicb.2018.00959 (PMC5962736; doi:10.3389/fmicb.2018.00959)

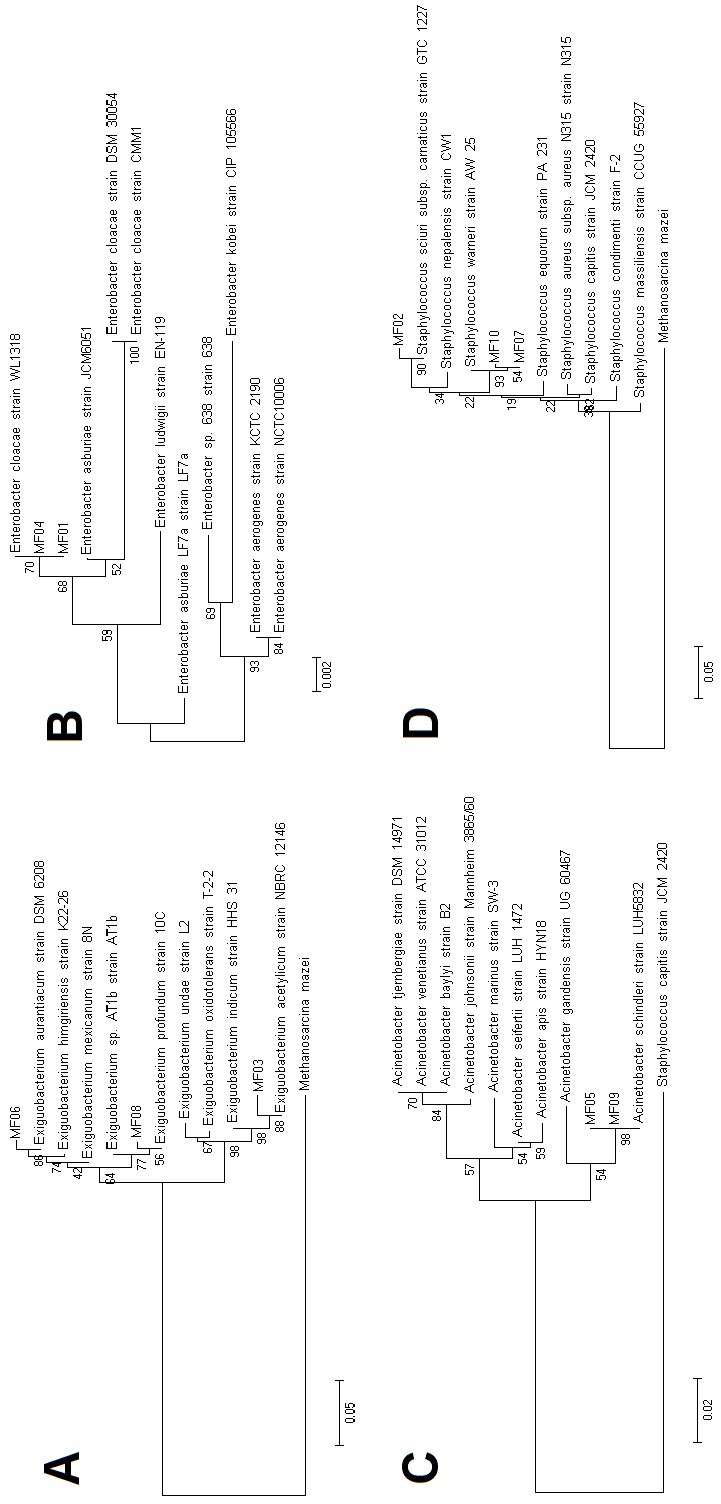

Supplement: Figure S1 — Phylogenetic trees based on the 16S rRNA gene sequence. Trees were constructed using the neighbor-joining method as described in Methods. (A) Exiguobacterium, (B) Enterobacter, (C) Acinetobacter, and (D) Staphylococcus. The numbers on the nodes represent the percentage of bootstrap (1,000 repetitions). [file Image_1.JPEG]

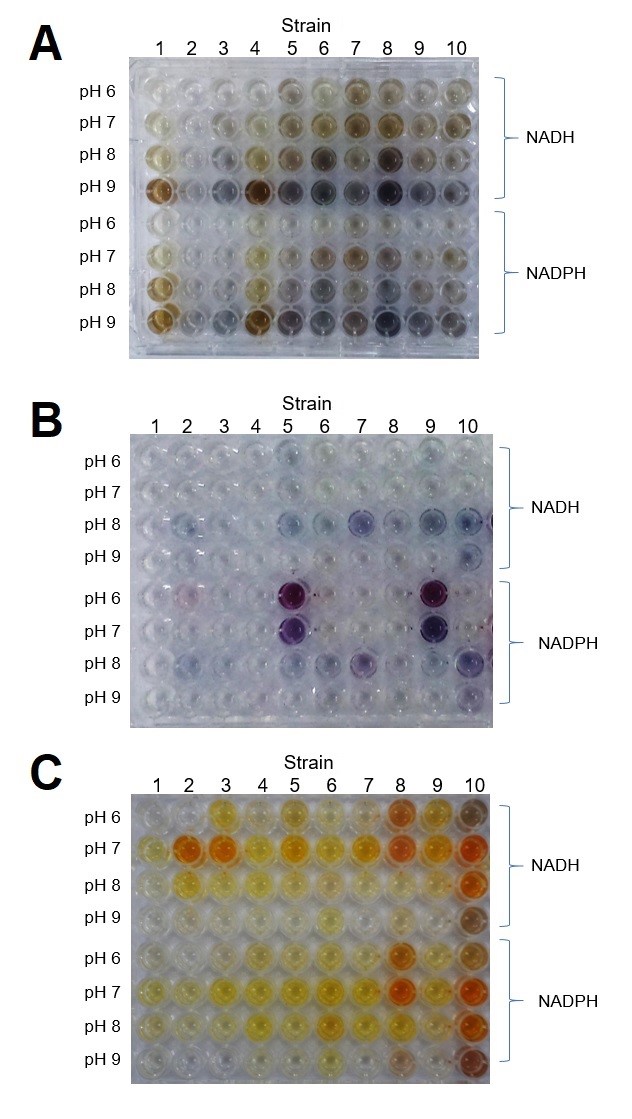

Supplement: Figure S2 — in vitro metal(loid)- reduction by crude extracts of the indicated strains. The reduction of TeO32-(A), AuCl4-(B), and Ag+(C) was performed as described in Methods. The activity was measured at different pH values and monitored for 24 h. Strains analyzed: (1) MF01, (2) MF02, (3) MF03, (4) MF04, (5) MF05, (6) MF06, (7) MF07, (8) MF08, (9) MF09, (10) MF10. [file Image_2.JPEG]

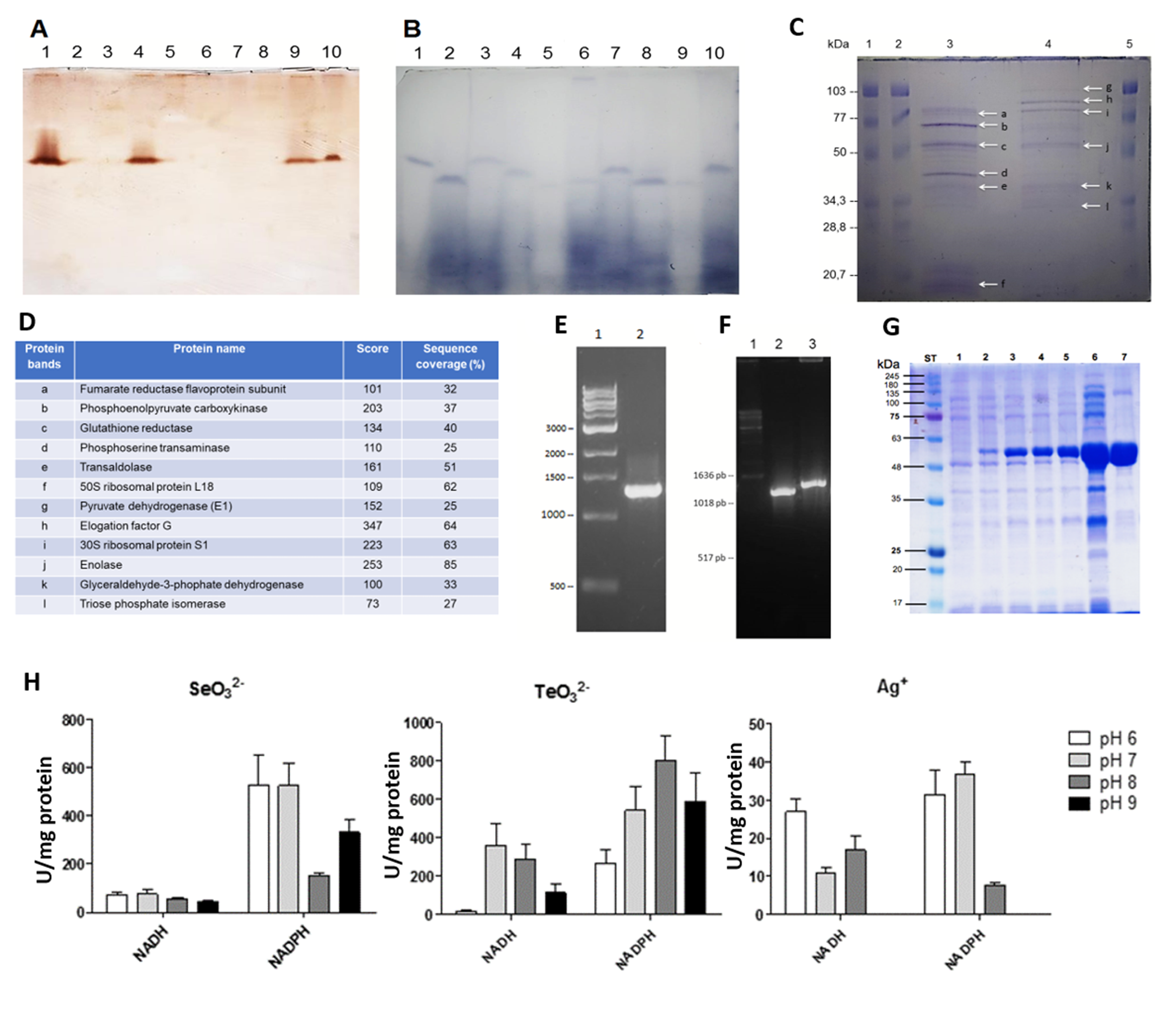

Supplement: Figure S3 — Identification, cloning, and purification of GorA. Tellurite (A) and gold (B) reductase activity in situ. Crude extracts from each strain [MF01 (1), MF02 (2), MF03 (3), MF04 (4), MF05 (5), MF06 (6), MF07 (7), MF08 (8), MF09 (9), MF10(10)] were fractionated by NATIVE-PAGE and revealed as TeO32- or AuCl4- activity as described in Methods. (C) SDS-PAGE of the reduction bands of TeO32- and AuCl4- from MF01 extracts; Lanes 1, 2, and 5: molecular weight standards (BioRad, #1610305). Lanes 3 and 4: fractionation of proteins present in the reduction band of TeO32- and AuCl4-, respectively. (D) Identification of protein bands by MALDI-TOF. (E) Agarose gel electrophoresis of PCR-amplified gorA; Lane 1, molecular size standards (1 kb DNA ladder, Promega). Lane 2, amplification of gorA. (F) Agarose gel electrophoresis of gorA amplification by PCR. Lane 1, molecular size standards (1 kb DNA ladder, Invitrogen); lane 2, colony PCR amplification of gorA; lane 3, directional cloning of gorA assessed by PCR using the primers Gforward and pETReverse described in section Growth, identification and characterization of the environmental strains; (G) Induction kinetics and purification of GorA; SDS-PAGE of crude extracts of E. coli overexpressing gorA and the purified enzyme; ST, SDS-PAGE molecular weight standards (low range). Lanes 1-6, induction kinetics for GorA at 0, 1, 2, 3, 4, and 16 h, respectively. Lane 7, purified recombinant GorA. (H) GorA metal(loid)-reductase activity (U/mg protein), showing the dependence on the pH and cofactor. [file Image_3.TIF]

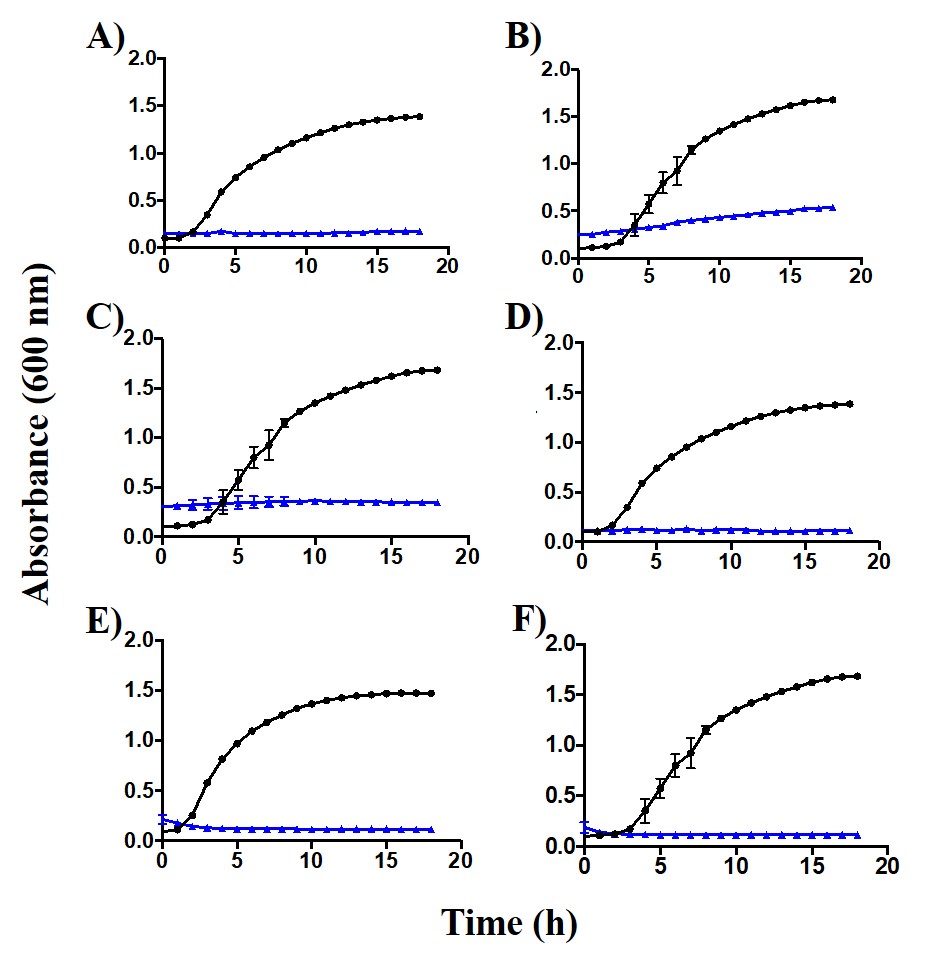

Supplement: Figure S4 — Growth curves of E. coli and L. monocytogenes exposed to NS. Effect of TeNS synthesized by crude extracts of cells overexpressing the E. cloacae gorA gene in E. coli (A) and L. monocytogenes (B). Effect of TeNS synthesized using crude extracts of MF09 in E. coli (C) and L. monocytogenes (D). Effect of AuNS synthesized by crude extracts of MF09 in E. coli (E) and L. monocytogenes (F). Black and blue lines represent the growth of control and NS-treated cells, respectively. [file Image_4.JPEG]
