# Supplementary material for: Synthesis and Antibacterial Activity of Metal(loid) Nanostructures by Environmental Multi-Metal(loid) Resistant Bacteria and Metal(loid)-Reducing Flavoproteins
Source: Front Microbiol. 2018 May 15;9:959. doi: 10.3389/fmicb.2018.00959 (PMC5962736; doi:10.3389/fmicb.2018.00959)
Supplement: Table S1 — Metal(loid)-resistant strains. [file Table_1.docx]

Supplementary Material

Synthesis and Antibacterial Activity of metal(loid) nanostructures by environmental multi-metal(loid) resistant bacteria and metal(loid)-reducing flavoproteins

Maximiliano Figueroa^1#^, Valentina Fernandez^1#^, Mauricio Arenas-Salinas^2^, Diego Ahumada^1^, Claudia Muñoz-Villagrán^1,3^, Fabián Cornejo^1^, Esteban Vargas^4^, Mauricio Latorre^5,6,7,8^, Eduardo Morales^9^, Claudio Vásquez^1^ and Felipe Arenas^1^*

*** Correspondence:** Felipe Arenas: [felipe.arenass@usach.cl](mailto:felipe.arenass@usach.cl)

**Table S1. Metal(loid)-resistant strains.**


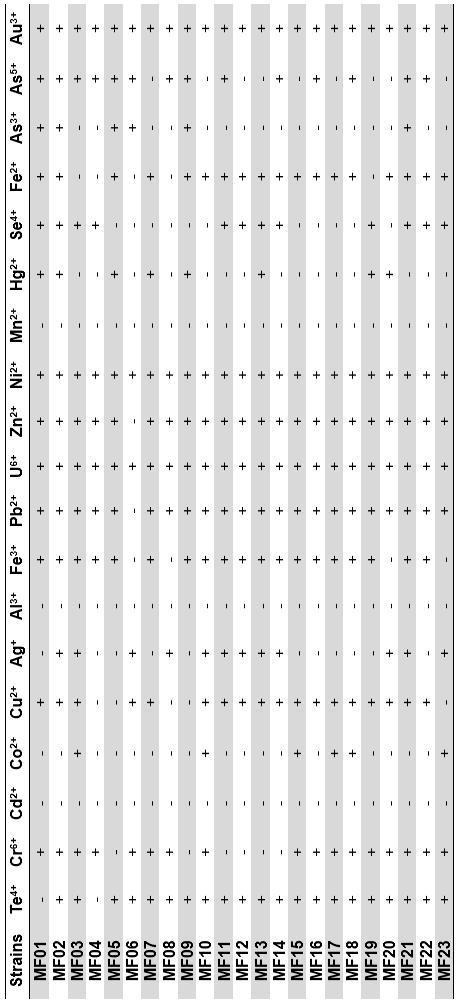


(+) growth, (-) no growth.

**Table S1 (continuation). Metal(loid)-resistant strains.**


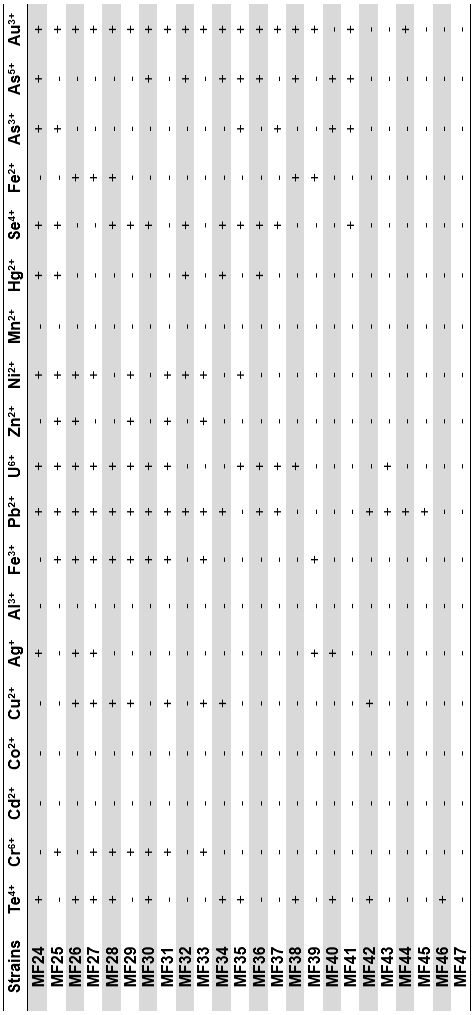


(+) growth, (-) no growth.
