# Supplementary material for: Synthesis and Antibacterial Activity of Metal(loid) Nanostructures by Environmental Multi-Metal(loid) Resistant Bacteria and Metal(loid)-Reducing Flavoproteins
Source: Front Microbiol. 2018 May 15;9:959. doi: 10.3389/fmicb.2018.00959 (PMC5962736; doi:10.3389/fmicb.2018.00959)
Supplement: Table S2 — MICs for the metal(loid)-resistant strains. [file Table_2.docx]

Supplementary Material

Synthesis and Antibacterial Activity of metal(loid) nanostructures by environmental multi-metal(loid) resistant bacteria and metal(loid)-reducing flavoproteins

Maximiliano Figueroa^1#^, Valentina Fernandez^1#^, Mauricio Arenas-Salinas^2^, Diego Ahumada^1^, Claudia Muñoz-Villagrán^1,3^, Fabián Cornejo^1^, Esteban Vargas^4^, Mauricio Latorre^5,6,7,8^, Eduardo Morales^9^, Claudio Vásquez^1^ and Felipe Arenas^1^*

*** Correspondence:** Felipe Arenas: [felipe.arenass@usach.cl](mailto:felipe.arenass@usach.cl)

**Table S2. MICs for the metal(loid)-resistant strains**

|  |  | **MIC (mM)** | | | | | | | | |
| --- | --- | --- | --- | --- | --- | --- | --- | --- | --- | --- |
|  | **Strain** | **TeO_3_^2-^** | **AuCl_4_^-^** | **Ag^+^** | **AsO_2_^-^** | **CrO_4_^2-^** | **Cu^2+^** | **Cd^+^** | **SeO_3_^2-^** |  |
| **MF01** | *Enterobacter cloacae* | 0.008 | 0.25 | 0.5 | 3.125 | 3.125 | 12.5 | 2 | 500 |  |
| **MF02** | *Staphylococcus sciuri* | 4 | 0.25 | 0.25 | 25 | 400 | 6.25 | 1 | 500 |  |
| **MF03** | *Exiguobacterium acetylicum* | 0.25 | 0.25 | 0.3 | 3.125 | 12.5 | 6.25 | 1 | 250 |  |
| **MF04** | *Enterobacter cloacae* | 0.001 | 0.25 | 0.25 | 25 | 0.78125 | 6.25 | 1 | 62.5 |  |
| **MF05** | *Acinetobacter schindleri* | 1 | 0.25 | 0.25 | 25 | 3.125 | 6.25 | 1 | 31.2 |  |
| **MF06** | *Exiguobacterium aurantiacum* | 0.25 | 0.125 | 0.0625 | 25 | 100 | 1.56 | 1 | 6.25 |  |
| **MF07** | *Staphylococcus warneri* | 0.5 | 0.5 | 0.25 | 12.5 | 50 | 3.75 | 1 | 6.25 |  |
| **MF08** | *Exiguobacterium profundum* | 0.5 | 0.25 | 0.25 | 25 | 400 | 3.12 | 1 | 250 |  |
| **MF09** | *Acinetobacter schindleri* | 1 | 0.25 | 0.25 | 25 | 6.25 | 3.12 | 1 | 250 |  |
| **MF10** | *Staphylococcus warneri* | 1 | 0.25 | 0.25 | 12.5 | 200 | 6.25 | 0.5 | 3.125 |  |
|  |  |  |  |  |  |  |  |  |  |  |
